# Aqueous mounting media increasing tissue translucence improve image quality in Structured Illumination Microscopy of thick biological specimen

**DOI:** 10.1038/s41598-018-32191-x

**Published:** 2018-09-18

**Authors:** Aleksander Szczurek, Fabio Contu, Agnieszka Hoang, Jurek Dobrucki, Sabine Mai

**Affiliations:** 1 University of Manitoba, Cancer Care Manitoba, Winnipeg, 675 McDermot Ave, R3E 0V9 Canada; 2 Department of Cell Biophysics, Faculty of Biochemistry, Biophysics and Biotechnology, Jagiellonian University, Krakow, Gronostajowa 7, 30-387 Poland; 3 University of Cagliari, Unit of Biology and Genetics, Department of Biomedical Sciences, S. P. Monserrato, Sestu Km 0.700, Cagliari, 09042 Italy

## Abstract

Structured Illumination Microscopy (SIM) is a super-resolution microscopy method that has significantly advanced studies of cellular structures. It relies on projection of illumination patterns onto a fluorescently labelled biological sample. The information derived from the sample is then shifted to a detectable band, and in the process of image calculation in Fourier space the resolution is doubled. Refractive index homogeneity along the optical path is crucial to maintain a highly modulated illumination pattern necessary for high-quality SIM. This applies in particular to thick samples consisting of large cells and tissues. Surprisingly, sample mounting media for SIM have not undergone a significant evolution for almost a decade. Through identification and systematic evaluation of a number of non-hazardous, water-soluble chemical components of mounting media, we demonstrate an unprecedented improvement in SIM-image quality. Mounting solutions presented in this research are capable of reducing abundant light scattering which constitutes the limiting factor in 3D-SIM imaging of large Hodgkin’s lymphoma and embryonic stem cells as well as 10 µm tissue sections. Moreover, we demonstrate usefulness of some of the media in single molecule localisation microscopy. The results presented here are of importance for standardisation of 3D-SIM data acquisition pipelines for an expanding community of users.

## Introduction

Structured Illumination Microscopy (SIM) is a fluorescence-based super-resolution microscopy method providing isotropic resolution improvement by a factor of 2 over diffraction-limited microscopy^1,2^. It has been broadly recognised in cell biology research and applied in various studies including investigations of architecture of the cell nucleus^3,4^, DNA repair processes^5^, applied to microbiology^6^ and many others.

SIM relies on structured illumination (fringe) patterns generated using linear transmission phase grating^2^, interferometer^7^, or using a spatial modulator^8^. In SIM the illumination pattern is transmitted through a high numerical aperture (NA) objective in several orientations and phases and is projected further onto a fluorescently labelled sample. Consequently, high spatial frequencies representing the sample become combined with illumination pattern frequency making them transmittable through the objective lens (analogically to “Moiré fringes”). In the process of back-calculation, the sample’s spatial frequencies, that are invisible to conventional microscopy, are extracted in Fourier space and transformed into image space to form a super-resolution SIM image. The process is repeated at various depths within the sample in order to capture its 3D features.

High-resolution SIM relies on raw image data with high-quality point spread function and high signal-to-noise ratio. Consequently, highly contrasted illumination patterns in the imaged location of a sample are desired. These aspects make SIM extremely sensitive to various inherent sample imperfections including refractive index (RI) discontinuity leading to scattering, spherical aberrations, and low photon numbers^9^. Such conditions commonly occur in biological samples containing various scattering structures such as lipid droplets, lipid bilayer interfaces^10^, tightly packed proteins in nucleoli, or cell nuclei^11^.

Various methods have been proposed to overcome the above limitations^12^, many relying on complex hardware add-ons. For instance wave-front was measured in scattering media and corrected with feedback-based algorithms to produce close to ideal SIM illumination patterns adapted to inherent sample imperfections^13^. This approach enabled correction of the aberrations and restoration of the highly modulated illumination pattern within biological samples^14^. Experimentally, RI mismatch between the immersion oil and the sample’s mounting medium has been minimised through adjustment of the objective’s correction collar or via immersion oils of a refractive index slightly deviating from the one of glass (RI_glass_ = 1.518) applied on the objective. In the latter, immersion oils of RI ranging from 1.512–1.530 were used, dependent upon sample thickness^15^. This approach, however, does not aim to reduce the effects of light scattering at refracting interfaces but rather to compensate for an overall mismatch between RI of the mounting medium and glass.

Recently, the field of tissue clearing has gained increasing attention^16^. Optical clearing is intended to diminish local refractive index inhomogeneities by facilitating penetration of mounting media of RI close to 1.5 into thick biological samples and even whole organs. This allows to more precisely match the RI of the sample with microscopy optics, reduce RI variation across the sample, and therefore diminish light refraction and scattering on cellular structures. This approach leads to a decrease in effects associated with spherical aberration and enables fluorescence imaging of biological samples at a depth of up to several millimeters^17^. Although SIM has been mostly used along with commercially available anti-fading media with RI of 1.45^3^, the first attempts to apply optical tissue clearing in SIM have already demonstrated image quality improvements^18^.

In this work, we present a simple solution to the problem of refractive index discontinuities and provide ready-to-use protocols for sample mounting for 3D-SIM simplifying the process of data acquisition and providing increased super-resolution image quality. Protocols with a total workload under 1 hour utilise non-hazardous reagents and are based primarily on aqueous solutions. Using publically available software^19^ and a commercial SIM set-up we quantitatively compare the quality of raw SIM data recorded using popular sample mounting procedures and our new protocols. Moreover, we demonstrate that photo-stability of fluorescent probes commonly used in multicolour SIM is not compromised with the new SIM clearing mounting media presented here. Finally, we explore the usefulness of our novel mounting media in single molecule localisation (SMLM) and correlative super-resolution microscopy. This work is relevant to previous efforts to standardise experimental SIM protocols among the growing community of users^19–22^.

## Results

### Hodgkin’s lymphoma – a challenging sample for 3D-SM imaging

Most of the previous SIM studies utilised adherent cells, i.e. a very convenient model due to their thickness of only ~3 µm. However, many cell types and whole tissues exhibit more complex shapes and require imaging throughout larger distances into the sample. Importantly, SIM quality is significantly impaired when imaging is performed further away from the coverglass, e.g. approx. 10 µm deeper into a sample. In order to investigate difficulties associated with SIM imaging at this depth we imaged Hodgkin’s lymphoma (HL) cells for the following reasons: (i) they have a globular, complex shape and grow in suspension, (ii) they are often multinucleated and (iii) have a large thickness of >10 µm. The nuclear DNA was stained using Hoechst 33258 DNA-binding dye. This dye was chosen for its short excitation and emission wavelengths that are refracted more than longer visible wavelengths. In addition, these wavelengths are highly absorbed by biological material, making them especially challenging in imaging thick samples^23^. On the other hand, fluorescent probes emitting short wavelengths of light are capable of providing the highest resolution in SIM (approx. 100 nm laterally and 350 nm axially).

We first evaluated the performance of 3D-SIM in cell samples embedded in Vectashield, as it is the most commonly used non-hardening mounting medium recommended for SIM^22,24^. Due to its refractive index only moderately matching the one of the optics (1.448 vs. 1.518) a correction routine has been proposed. SIM data for several objective immersion oils with various RI are visually inspected and the best performing one is incorporated into further experiments^15,21^. We tested this protocol and embedded the HL samples in Vectashield and performed 3D-SIM using a set of immersion oils ranging from RI 1.510 to 1.518. In pursuance of a minimum researcher evaluation bias and in order to quantitatively determine the quality of raw 3D-SIM data in whole cells we measured the average modulation contrast-to-noise ratio (MCNR)^19^. MCNR serves as a convenient parameter reflecting SIM stripe contrast of structural features in the image plane of raw 3D data. Low MCNR values (i) indicate a low fluorescence signal-to-noise ratio of the raw data, or (ii) indicate a high level of contamination with unmodulated background (e.g. by autofluorescence or out-of-focus blur), or (iii) correspond to fluorescence images with blurred SIM illumination pattern-driven fringes (e.g. due to spherical aberration obscuring the ideal optical transfer function of the microscope (the Fourier Transform of point spread function) resulting in specific artefacts^12,21^). High MCNR values coincide with a high contrast and rather obvious SIM pattern required for imaging beyond the diffraction limit of the resolution^19^ (for examples see Supplementary Fig. S1). MCNR values below 4 are considered inadequate for meaningful SIM, values between 4–8 correspond to low to moderate raw SIM data quality, and values >8 are considered good^19^.

**Figure 1:**
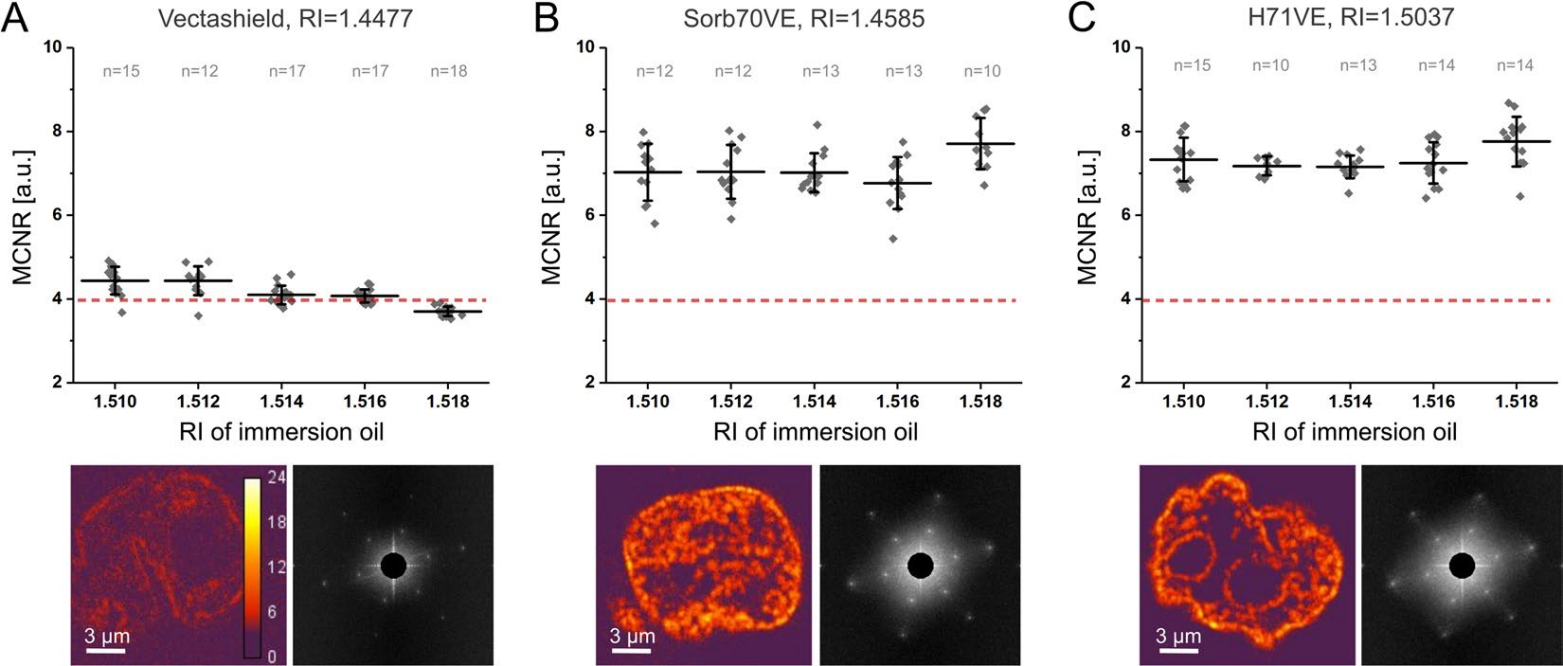
Quantitative comparison of raw SIM data quality. (**A**) MCNR calculated for Hodgkin’s lymphoma cells stained for the DNA with Hoechst 33258 and embedded in Vectashield. The red dashed line indicates an MCNR value threshold equal to 4 below which the data do not pass the evaluation and are not acceptable for SIM^19^. Each data-point represents a single cell measurement. Median and standard deviation values indicated in black. Below: example of MCNR maps and corresponding frequency spectra for raw SIM image sequences. Analogous data for Sorb70VE and H71VE media in (**B**) and (**C**), respectively. Low frequencies of high abundance are hidden behind a circular mask for clarity of data presentation. Note the strikingly different FT spectrum and MCNR map for Vectashield. Examples of FT spectra and MCNR maps for single cells in different media (images acquired with 1.518 immersion oil). All measurements were statistically different from respective measurements carried out in Vectashield, i.e. p-value in t-test <0.001. See Supplementary Fig. S4 for the same experiment for F80VE and S65VE media. Fluorescence signal dynamic range is compared in Supplementary Table 3.

While most of the immersion oils tested on Vectashield-embedded specimen scored MCNR values close to 4, i.e. the lowest acceptable values for meaningful SIM^19^, introduction of a 1.510 and 1.512 RI immersion oils resulted in median MCNR equal to 4.50 +/− 0.33 and 4.51 +/− 0.35 respectively (Fig. 1A). Comparable outcomes were also obtained for DAPI-stained HL cells (Supplementary Fig. S2). These values, although the highest achieved in this assay, are much lower than the ones reported for flat or small cells, that is in healthy human white blood cells whose MCNR amounts to 10.25 +/− 0.43 (Supplementary Fig. S1). We find the following explanation of low MCNR values, corresponding to low pattern quality (Supplementary Fig. S3) in HL cells - the immersion-oil based adjustment aims at matching the optical properties of the sample (in the region of interest) with the properties encoded in the optical transfer function (OTF) used for the reconstruction. However, the cells contain many constituents such as lipids, protein aggregates, chromatin or dense nucleoli of RI higher than that of the mounting medium applied (1.448) but closer to the RI of glass^10,25^. As a consequence, illumination pattern as well as fluorescence originating from a sample undergo scattering. Its negative effects on the raw image, the structured illumination quality and the MCNR value could not be ultimately reduced with the aforementioned oil-based correction (Fig. 1A). Moreover, these effects are more pronounced in thicker specimens as they are proportional to a length of the light path through a scattering medium.

### 3D-SIM quality improvement by sample mounting in different media

As an alternative approach, we set out to reduce differences in refractive index underlying light scattering on cellular constituents by screening mounting media with RI as close to 1.518 (RI_glass_) as possible. We assumed that the new media for SIM need to fulfil the following additional criteria: (i) the media should not desiccate the sample and interfere with the three-dimensional structure of the cells, (ii) the media should readily penetrate the sample, yielding homogenous RI across specimen, and finally (iii) the media should minimise photobleaching and provide high signal-to-noise ratio throughout 3D data collection. Similar criteria are applicable to media used previously for whole organ clearing (reviewed in Richardson *et al*.^16^). We reasoned that the incorporation of such an approach would yield a twofold benefit: minimise light refraction on sample features and improve the resolution of a microscope. Consequently, we expected that the fringes corresponding to SIM illumination pattern detected in fluorescence patterns in raw SIM images would be maximised.

The water-soluble agents we evaluated were sucrose, sorbitol, fructose, and iohexol (commercial name Histodenz). All of these agents at high concentrations are characterised by relatively high RI ranging from 1.46–1.52 and have been previously shown to be useful in whole-organ clearing^17,26–28^. These agents alone however, in contrast to Vectashield, do not have a capacity to minimise fluorophore photobleaching. Therefore, we supplemented the mounting media based on the aforementioned water-soluble agents with a 20% vol/vol addition of Vectashield (see Supplementary Table 1 for complete protocols).

In the next step we assessed the effect of immersion oils with varying RI on the overall MCNR value for HL cell samples embedded in various media. The first mounting medium was based on a 70% wt/wt sorbitol with an addition of 20% vol/vol Vectashield in PBS and the final RI was comparable to Vectashield (RI~1.46, further abbreviated as Sorb70VE). The second mounting medium comprised 71.4% wt/wt Histodenz mixed with 20% vol/vol Vectashield in PBS (final RI ~1.50, further called as H71VE medium).

In this experiment the highest MCNR values were obtained for the immersion oil with RI = 1.518: median MCNR for Sorb70VE and H71VE equals to 7.59 +/− 0.62 and 7.91 +/− 0.59, respectively (Fig. 1B,C). These values are significantly greater than the highest achievable value in the analogous experiment for HL cells embedded in Vectashield (Fig. 1A). Moreover, frequency spectra for cells embedded in Sorb70VE and H71VE, unlike in the case of Vectashield, reveal pronounced peaks corresponding to the frequency of the SIM illumination pattern (Fig. 1, bottom) further supporting a notion of enhanced raw SIM illumination pattern quality (see Supplementary Fig. S3 for examples).

**Figure 2:**
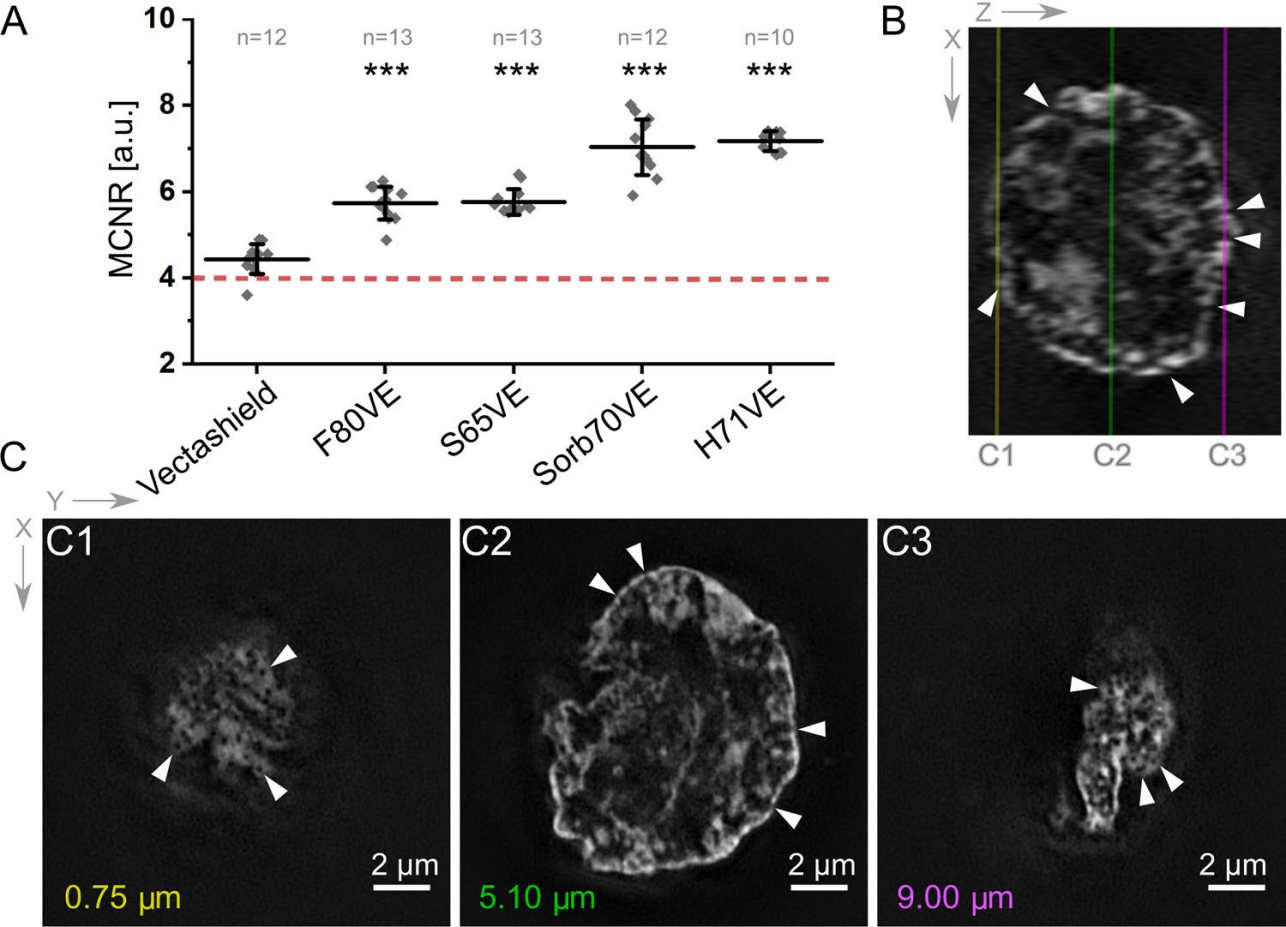
Evaluation of 3D-SIM data acquired in various mounting media. (**A**) MCNR values scored for raw 3D-SIM data of Hodgkin’s lymphoma cells embedded in various mounting media. DNA stained with Hoechst 33258 was imaged. The red dashed line indicates MCNR value threshold below which the data are not acceptable for meaningful SIM^19^. Each data-point corresponds to a single cell 3D-SIM measurement. The total number of cells measured indicated on top in gray (n). Measurements acquired with 1.512 immersion oil. All measurements were statistically different from respective measurements carried out in Vectashield (p-value <0.001). (**B**) Orthogonal view (XZ) of a 3D-SIM reconstruction of a HL cell embedded in H71VE medium with marked horizontal cross-sections (lines, C1–C3). (**C**) Horizontal (XY) views from different imaging depths through a cell: C1, bottom; C2, middle; C3, top. Approximate respective distances from the coverslip (imaging depth) are indicated. Arrowheads in B and C1–C3 point at nuclear periphery where openings between chromatin domains and entries into the interchromatin compartment occur. Scale bars corresponding to 2 µm apply to all images presented in B and C.

Encouraged by these results, we further compared MCNR values for other agents prepared in a similar way, i.e. based on sucrose (S65VE) and fructose (F80VE) (Supplementary Table 1). All mounting media tested in our assay scored relatively high median MCNR values for 1.512 immersion oil (Fig. 2A): 5.63 +/− 0.29 for S65VE and 5.63 +/− 0.29 for F80VE (see Supplementary Fig. S4 for more details). Importantly, all mounting media scored much higher MCNR values than Vectashield with immersion oil-based compensation (Fig. 1A)^15,21^, demonstrating that the solution presented in this work provides a superior alternative for this time consuming adjustment. To rule out the effect of difference between signal intensities we also performed a similar 3D-SIM experiment on thick mouse embryonic stem cells using excitation and detection parameters yielding maximal dynamic range of signal intensity for each measurement (Supplementary Fig. S5).

3D-SIM reconstructions of HL cells embedded in SIM-dedicated mounting media based on the strategy of optical tissue clearing result in a high level of detail. Round and sharp outlines of the cell nucleus are clearly noticeable in all orientations when cells are embedded in S65VE, Sorb70VE, and H71VE (Fig. 2B). Chromatin at the nuclear periphery harbours evenly distributed circular regions devoid of DNA staining with diameters of <190 nm, that have been shown to correspond to the positions of nuclear pore complexes^29^, and remain unresolvable by means of conventional microscopy^3^. In contrast, SIM imaging of HL cells embedded in standard Vectashield mounting medium could not resolve nuclear periphery structures and was limited by SIM artefacts (Supplementary Fig. S6). We attribute this poor outcome to the low modulation contrast of the SIM pattern (Supplementary Fig. S3).

### 3D-SIM in tissue sections embedded using SIM-dedicated mounting protocols

**Figure 3:**
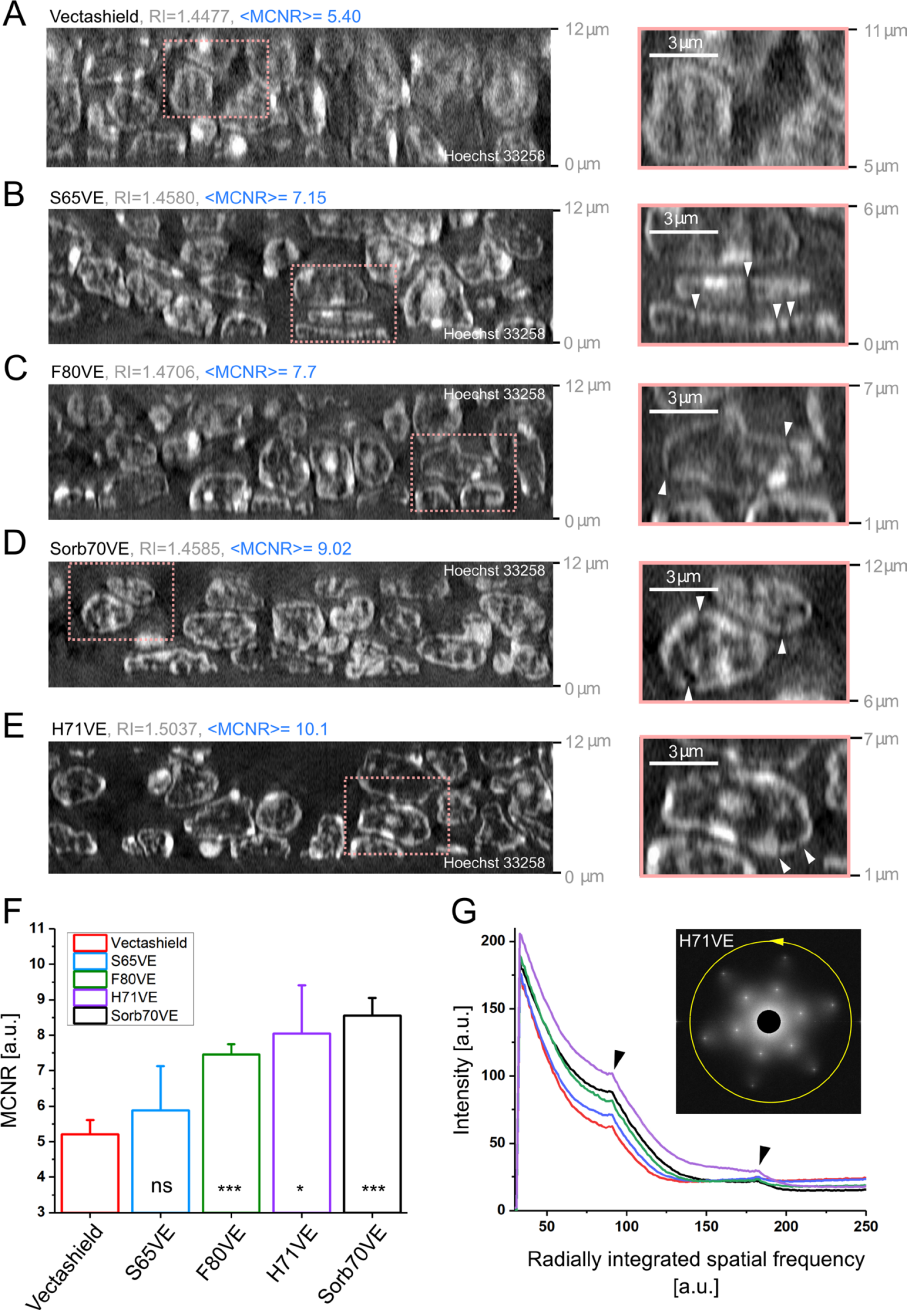
3D-SIM imaging of mouse spleen tissue sections. 10 µm-thick tissue sections were stained for nuclear DNA using Hoechst 33258. Subsequently, they were embedded in different mounting media in order to equalise its optical properties, decrease light scattering, and improve quality of 3D-SIM images. 12 µm-thick orthogonal cross-sections (XZ) through 3D-SIM images demonstrate axial and horizontal resolution achieved in specimen embedded in Vectashield (**A**), S65VE (**B**), F80VE (**C**), Sorb70VE (**D**) and H71VE (**E**). 0 µm corresponds to an imaging plane closest to the coverslip. For the sake of clarity the values of respective refractive indices and MCNR are given. Enlarged insets are provided on the right-hand side. Scale bar equals to 3 µm. White arrowheads in B–E indicate interruptions in the nuclear periphery unravelled due to enhanced resolution. Intensity histogram was adjusted to min-max. (**F**) Bar-plot presenting median values of MCNR scored for various clearing media (at least 3 measurements). Error bars correspond to the standard deviation. (**G**) Graph demonstrates radial profiles of averaged frequency spectra obtained for several raw 3D-SIM images. Same colour coding was used as in F. Original frequency spectra provided in Supplementary Fig. S6A. Inset presents an example of averaged frequency spectrum obtained for mouse spleen sections embedded in H71VE. Yellow arrow demonstrates the direction of intensity integration. Black arrowheads point at peaks corresponding to SIM illumination pattern frequencies.

Next, we performed 3D-SIM on 10 µm-thick paraffin-embedded mouse spleen tissue sections optically cleared with new mounting media dedicated for SIM. Figure 3A–E demonstrates orthogonal views through 3D-SIM images of mouse spleen sections embedded in TDE97, Vectashield, S65VE, F80VE, Sorb70VE, and H71VE.

The highest MCNR values were achieved for Sorb70VE, H71VE and F80VE (Fig. 3F). This was further reinforced by radially integrated frequency spectra (Fig. 3G). Here the most pronounced peaks corresponding to the SIM illumination pattern frequencies were observed for the same media. In this type of experiment we expect that it is important to control the distance *d* between a tissue section (placed on a glass slide) and a coverslip. Larger *d* and lower RI values of the mounting medium likely introduce spherical aberration leading to an ultimate deterioration of the illumination pattern quality^12^. Indeed MCNR values for media of the highest RI are significantly higher (Fig. 3F). A similar phenomenon is evident from frequency spectra; the peaks corresponding to frequency of the illumination pattern are most pronounced for these mounting media (Fig. 3G, black arrowheads, Supplementary Fig. S7A). However, profiling of MCNR values across the imaging depth within a tissue revealed that the data acquired in F80VE and Sorb70VE show a 23% and 18% drop in MCNR value across ~10 µm imaging depth as compared to <10% for other media (Supplementary Fig. S7B-C). MCNR remains constant across the entire imaging depth for H71VE-embedded sections as well. This implies a relatively even 3D resolution in the entire 3D-SIM images which is desired in super-resolution microscopy imaging.

Orthogonal views through mouse spleen sections embedded in SIM-dedicated mounting media allow a clear separation of individual nuclei in 3D, in contrast to samples embedded in Vectashield (Fig. 3B–E). Individual chromatin domains and interchromatin compartments located adjacent to the nuclear periphery are certainly visible in all orientations in 3D-SIM images for S65VE, F80VE, H71VE, and Sorb70VE (Fig. 3B–E, insets, arrowheads)^4^.

Subsequently, we attempted to perform 3D-SIM measurements on 20 µm-thick mouse spleen sections using H71VE mounting medium, as it performed very well in our previous evaluation. The experiment revealed that MCNR amounted to 5.79 in comparison to 7.70 +/− 1.36 obtained for 10 µm-thick sections prepared in a similar fashion (Supplementary Fig. S8). Interestingly, MCNR values were relatively low across all imaging depths when compared to 10 µm-thick sections (Fig. 3F). A relatively low MCNR value resulted in a characteristic impairment of axial resolution in 3D-SIM reconstructions. As a consequence the sectioning capability of 3D-SIM became compromised. This was reflected by a distinctive axial smear of the signal below and above nuclei.

### Fluorescence preservation

Until now our study relied on single-colour 3D-SIM measurements of Hoechst dye, which is relatively resistant to photobleaching. However, in molecular cell biology it is often relevant to study many fluorescently labelled targets simultaneously. Therefore, multicolour 3D-SIM appears to be absolutely essential. So far 3D-SIM using up to 3 colours has been demonstrated in adherent cell samples embedded in Vectashield^3^ that prevented fluorescence photobleaching during the time of exposure to an exciting light. Hence, we set out to study photobleaching kinetics of the fluorescent probes most commonly used in SIM including Hoechst 33258, Alexa488, Alexa555, Cy3, Alexa594, and Alexa647 followed by embedding in the new mounting media. Supplementary Figure S9 demonstrates a comparison of the kinetics of photobleaching for various fluorophores in different media. We noticed that almost all SIM-mounting media prevent fluorescence photobleaching well in comparison with standard Vectashield, in some cases even improving the brightness of some fluorophores (e.g. Alexa488 or Cy3 in S65VE). As a notable exception is H71VE medium containing Histodenz. This clearing agent supports high and stable fluorescent signal for Hoechst 33258 only, giving rather poor results for other dyes tested except Alexa555 and Cy3. We observed that fluorescence signal of Alexa594 was the most stable in all the media apart from H71VE in which the photobleaching proceeded rapidly. (Supplementary Fig. S9F,G). Moreover, we found that varying concentration of Vectashiled yields different anti-photobleaching protection of the media (Supplementary Fig. S10). Fluorescence signal intensity assessment using multicolour fluorescent beads as calibration objects also confirmed that H71VE medium provided a largely decreased signal to noise ratio whereas other media provide similar results to Vectashield (Supplementary Fig. S11). Furthermore, we demonstrate that 3-colour 3D-SIM in these media is feasible (Supplementary Fig. S12).

### SMLM in SIM-dedicated mounting media

Vectashield, which is routinely used to embed samples for 3D-SIM, has also been reported to facilitate photoswitching of Alexa647 with comparable photon counts to standard switching buffers based on thiols^30^. Since the SIM-dedicated mounting media investigated in this work contained 20% vol/vol Vectashield we set out to evaluate their usefulness in single molecule localisation microscopy (SMLM). We labelled nuclear lamina in adherent cells with an Alexa647-conjugated antibody, embedded them in different mounting media, and performed SMLM measurements. We decided to compare the integrated single molecule photoncounts and the average localisation uncertainty, as both reflect achievable resolution in SMLM.

**Figure 4:**
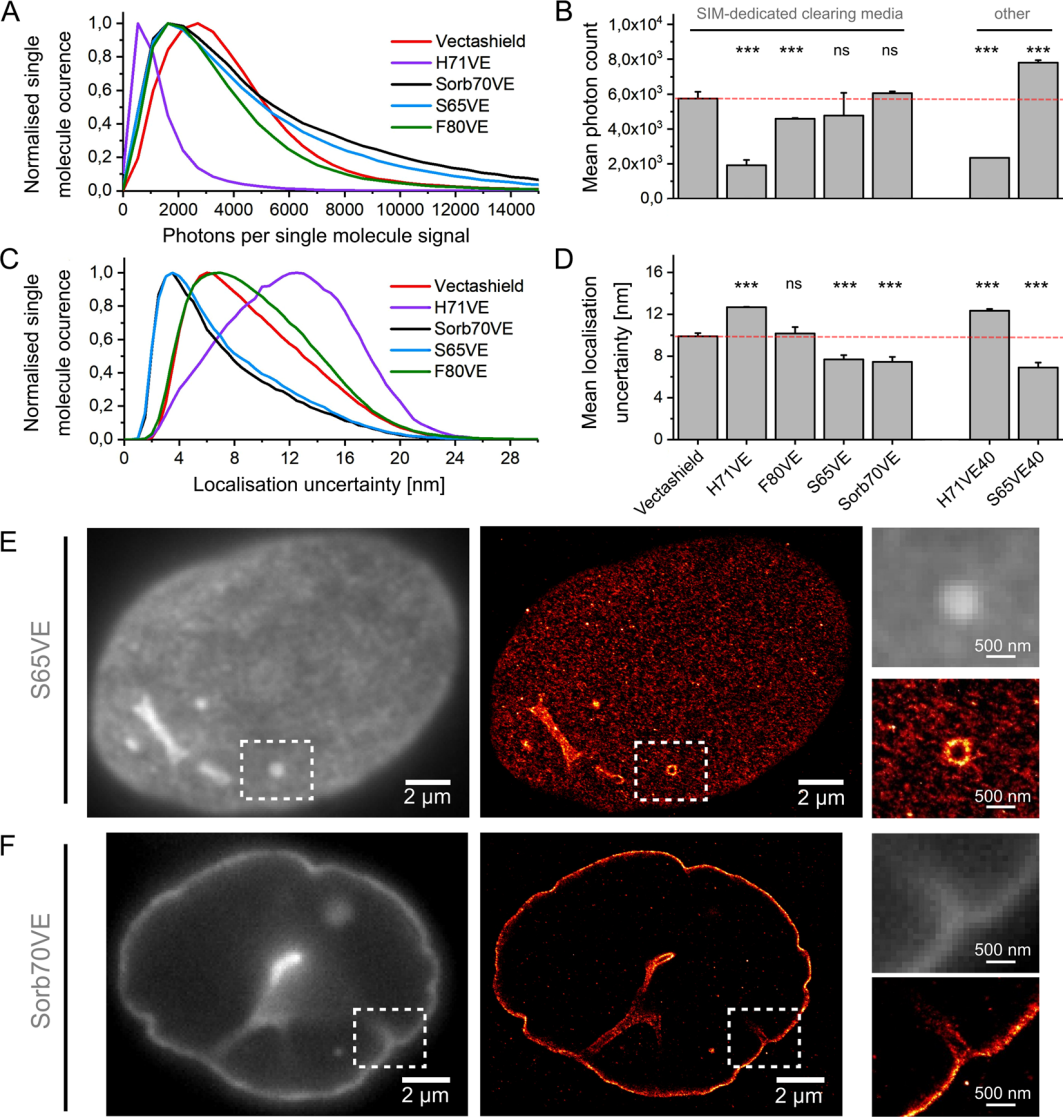
Single molecule localisation microscopy of Alexa647 in SIM-dedicated mounting media. A549 human pulmonary adherent cells were labelled for Lamin B1 using Alexa647-conjugated antibody and imaged under exposure to high intensity exciting light using a microscope suited for single molecule detection. (**A**) Collective histograms of photon counts for single molecule signals of Alexa647 acquired in 5 independent measurements (movies) after embedding in different SIM-dedicated mounting media (see Figs 1–3 for more details) with respective average values presented in a bar-plot in (**B**). See Supplementary Fig. S14 for respective data for standard SMLM mounting media based on cysteamine. (**C**) Collective histograms of corresponding localisation uncertainties with respective average values in (**D**). SIM-dedicated mounting media containing 20% vol/vol Vectashield were also compared to mounting media comprising 40% vol/vol mounting media (H71VE40, S65VE40 in B and D). See Supplementary Fig. S13 for respective transmitted light images of the cells embedded in a variety of media. (**E**,**F**) Examples of SMLM reconstructions of Lamin B1 – Alexa647 labelled A549 cells embedded in S65VE (**E**, nucleus bottom) and Sorb70VE (**F**, equatorial part of cell’s nucleus). Conventional wide-field images are presented in grey whereas SMLM reconstructions are colour-coded using a “red hot” look-up table. 2.5 × 3.0 µm^2^ region of interest (white dashed box) is enlarged and shown on the right-hand side of E and F.

As is evident from our quantitative evaluation, the performance of Alexa647 in SMLM after embedding in our set of mounting media dedicated for 3D-SIM of thick objects was comparable to the results achievable with routinely used Vectashield^30^ (Fig. 4). In particular, the most favourable values of localisation uncertainties were obtained for S65VE and Sorb70VE embedding media (Fig. 4A–D); here localisation uncertainties changed the distribution and reached average values of 7.7 +/− 0.5 nm and 7.4 +/− 0.3 nm, respectively. In contrast, H71VE medium provided only satisfactory results with an average photoncount approximately 3x lower comparing to Vectashield, and localisation uncertainty in the order of 12.6 +/− 0.6 nm. Further, increasing the Vectashield concentration from 20% vol/vol (H71VE) to 40% vol/vol (H71VE40) did not improve these values. Interestingly, a sucrose based medium supplemented with 40% vol/vol Vectashield (S65VE40) improved the brightness of single molecule signals by a factor of approximately 2, resulting in a very satisfying localisation uncertainty, in the order of 6.9 +/− 0.4 nm only vs. 9.9 +/− 0.4 nm for Vectashield (Supplementary Fig. S14). SMLM reconstructions of Lamin B1 obtained in SIM-dedicated media clearly demonstrate the resolution improvement over conventional wide-field microscopy; fine details of hollow tubular nuclear periphery invaginations became resolved in vertical (S65VE, Fig. 4E) and horizontal (Sorb70VE, Fig. 4F) orientations with respect to the imaging direction (see Supplementary Fig. S15 for results with the use of other media).

### Correlative SIM-SMLM

Knowing that the new mounting media gave excellent results with 3D-SIM (Figs 1–3) and allowed successful SMLM (Fig. 4) we investigated whether they enable correlative SIM&SMLM^7^. Such a strategy could provide the following advantages: (i) enable introduction of another high-resolution channel where SIM resolution improvement is insufficient (e.g. via using photo-switching of infrared probes such as Alexa647 on top of three other SIM channels), and (ii) help to identify and eliminate image reconstruction artefacts wherein the same structure is imaged with two different microscopy modalities^31^. Hence, we labelled nuclear lamina and DNA in healthy human white blood cells and attempted to image the first using 2D-SMLM (Alexa647) and subsequently the latter with 3D-SIM (Hoechst 33258) (Supplementary Fig. S16). Alexa647 signal in the presence of S65VE fluctuates strongly already under an exposure to relatively low excitation intensity (Supplementary Fig. S16, left column) allowing a super-resolution reconstruction using an algorithm based on radial fluctuations^32^. This approach permitted a successful 2D-SMLM reconstruction of nuclear lamina images with improved resolution and was followed by 3D-SIM imaging of the chromatin stained with Hoechst 33258 (Supplementary Fig. S16).

## Discussion

3D-SIM is a technique that relies on a well-defined illumination pattern, which, however, is sensitive to the slightest imperfections within an optical path resulting in optical aberrations. The specimen constitutes an integral part of the optical system as both exciting and fluorescence light travel through it and interact with it. However, the optical properties of a sample have not been sufficiently addressed in biological experiments utilising super-resolution microscopy and, to our knowledge, have been limited to only one report^18^. In particular, the quality of SIM illumination pattern strikingly degrades across the depth of imaging within thick specimens limiting meaningful SIM imaging to only a few µm within biological material^9,15^.

In this report, we fluorescently labelled the nuclear DNA in large Hodgkin’s lymphoma cells and in 10 µm-thick mouse spleen sections in order to highlight limitations of super-resolution 3D-SIM and proposed a solution to the problem of refractive index discontinuities and light scattering in these relatively large objects. We systematically compared the quality of raw 3D-SIM images obtained in aforementioned samples embedded in a variety of mounting procedures relying on solutions of sugars^17,26^, sugar alcohols^28^, and others^27,33^. Using new sample mounting procedures based on aqueous solutions with a high refractive index we attempted to reduce the effects associated with light scattering, and quantitatively compared the image quality improvement in raw 3D-SIM data.

Mounting the cell and tissue samples in Vectashield, the most commonly chosen imaging medium for 3D-SIM, gave results on the verge of acceptance even if the immersion oil–based compensation was introduced^15^ (Figs 1A and 3B). In contrast, HL cells and tissue sections embedded in the new mounting media presented in this work led to a considerable improvement in the quality of raw 3D-SIM data, in particular using H71VE, Sorb70VE, and S6570VE media (Figs 1B,C, 2 and 3B–E).

High quality SIM at large imaging depths of 20–30 µm was previously obtained with the help of deformable mirrors^14^. Further, SIM with multi-photon excitation has been successfully applied to imaging of thick nematode embryos^34^. These solutions, however, are rather expensive and remain inaccessible to most molecular cell biology laboratories that lack the advanced expertise in optics and typically use commercial SIM systems. Therefore, we hypothesised that the combination of the mounting media developed in this work with additional correction of aberrations using adaptive optics or using multi-photon excitation could enable high-quality SIM imaging at depths even greater than 30 µm. In this work we demonstrate that the clearing procedures alone are sufficient to enable high-quality 3D-SIM in 10 µm-thick mouse spleen sections (Fig. 3) and, to some extent, in 20 µm-thick mouse spleen sections (Supplementary Fig. S8). The poorer outcomes for 20 µm-thick sections might stem from considerable out-of-focus light as the structured illumination microscope used in this research operates in epi-illumination wide-field mode. This issue can be addressed in multiple ways, including differential imaging^14^ or changing an illumination scheme^35^.

All mounting media developed in this study resulted in improved quality of 3D-SIM images of thick specimens than for the previously used Vectashield^3^. Vectashield, however, is still a convenient medium for thin specimens. Interestingly, a significant quality improvement has been observed for S65VE and Sorb70VE, i.e. media with refractive index comparable to the one of Vectashield (1.45–1.46) pointing out that likely the high value of mounting medium’s RI is not the sole parameter crucial for precise clearing of biological specimens. We speculate that the viscosity of mounting media is at least as important as refractive index matching the system optics. Indeed, all the mounting media studied in this work are similarly or less viscous than Vectashield; this applies in particular to the intermediate clearing solutions (e.g. 30 to 60% wt/wt sucrose water solutions have viscosity of ~2 to 45 mPa·s^36^, see Supplementary Table 2 for details). Hence, these media can be expected to penetrate the specimen fairly rapidly leading to an enhanced uniformity of refractive index throughout a sample. In agreement with this line of argumentation the strongest loss of the average modulation to noise contrast (MCNR) across the depth of imaging in mouse spleen sections was observed for F80VE (Supplementary Fig. S7B,C), i.e. a mounting medium with the highest viscosity among all that were evaluated. In contrast, TDE97 in spite of its favourable refractive index (1.5149) and very low viscosity resulted in the worst performance (Supplementary Fig. S7A). We attribute this outcome, however, to the poor fluorescent signal of the fluorescent probes and very low signal-to-noise ratio in this medium^33^ (Supplementary Fig. S3). Further, high viscosity of some of the mounting media can be offset in part by elevating the temperature of the solutions while mounting the specimen. For instance the viscosity of glycerol (1410 mPa·s) can be decreased ~10 times when the temperature is raised from 20 to 50 °C.

To summarize, we recommend replacing Vectashield mounting medium for 3D-SIM experiments of 10 µm-thick tissue sections and large cells with H71VE, S65VE, or Sorb70VE depending on a set of fluorescent probes chosen (Supplementary Fig. S9, for summary see Table 1). These media are based on non-hazardous and easily accessible reagents and improve significantly the quality of raw 3D-SIM data by reducing sample opacity. We believe that simple sample preparation protocols presented in this work constitute another step forward in a continuing effort that the community of users has undertaken towards improvement and standardisation of SIM procedures^15,19–22^. Importantly, the mounting media dedicated for 3D-SIM of thick specimens can be further improved concomitantly with developments in the field of tissue clearing^16^. Further, S65VE, Sorb70VE, and F80VE media, due to their refractive index, are expected to be compatible with high-NA glycerol-immersed objectives^37^ and, at lower clearing agent concentrations, with silicon-immersed objectives. To our knowledge, such technical solutions have yet not been tested to improve 3D-SIM in thick specimens.

**Table 1:** Summary of the parameters of the mounting media tested and their performance in 3D-SIM of thick samples and SMLM.

Mounting medium for SIM:	Refractive Index @589 nm, 25 °C	Viscosity^a^	3D-SIM of thick samples					SMLM Alexa 647
			Hoechst 33258	Alexa 488	Alexa 555	Cy3	Alexa 594	
Vectashield	1.4477	− − − −	+	+	+	+	+	+++
S65VE	1.4580	− − −	+++	+++^c^*	++	+++^e^*	+++	+++^f^*
Sorb70VE	1.4585	− − −	++++*	+++	++*	+++^e^	+++*	+++*
F80VE	1.4706	− − − − −^b^	+++	++	++	+++^e^	+++	++
H71VE	1.5037	− − − −	++++	+^d^	+++^d^	++	+++^d^	+

Vectashield H-1000 was used in all experiments. Note a very low viscosity of intermediate solutions (Supplementary Table 2). Notes: ^a^Estimates of the viscosity of embedding media are provided for respective temperatures of embedding. See Supplementary Table 1 for more details. Viscosity scales proportionally with the number of “−” signs. ^b^High viscosity of fructose solutions can be decreased by an addition of urea^41^. This, however, may lead to DNA denaturation and impairment to Hoechst 33258 signal intensity. ^c^High signal to noise ratio with faster bleaching. ^d^Signal to noise ratio is low unless higher Vectashield vol/vol concentration in clearing media is used (Supplementary Fig. S10). ^e^Very high signal to noise ratio. ^f^Elevating the Vectashield concentration to 40% vol/vol improves Alexa 647 photoncount (see Supplementary Fig. S14). *Recommended medium for the fluorophore. Due to fluorescence signal instability in the presence of Vectashield Alexa 647 is not recommended in SIM.

Similarly to 3D-SIM, many other microscopy methods, including other super-resolution techniques, rely on high-quality 3D point spread function. We thus anticipate that other microscopy methods such as confocal laser scanning microscopy, light-sheet microscopy, Airy scan and single molecule localisation microscopy could also benefit from these new embedding protocols. Concerning the latter, in this report we demonstrate the utility of the SIM-dedicated media in photo-switching of a commonly used “blinking probe” Alexa647 (Fig. 4, Supplementary Figs S14–16). Thick biological specimens are typically imaged using multi-photon microscopy due to long imaging wavelengths of 650–1100 nm barely absorbed by biological material^34^. In this report however, we demonstrate a striking image quality improvement already using near-UV excitation (405 nm).

The importance of the new embedding procedures lies in their potential to expand the application palette of 3D-SIM beyond adherent cell monolayer to challenging super-resolution studies of various disorders where tissue sections are routinely utilised for diagnostic purposes. For instance, recent advancements demonstrated that 3D-SIM of tissue sections from patients has a potential to replace electron microscopy in diagnostics of glomerular histopathology^38^. Further, we speculate that the work presented in this article may help to facilitate advancing the super-resolution 3D-SIM studies from the monolayer cell culture models to multicellular spheroids or tissues.

## Materials and Methods

### Cell culture

Hodgkin’s lymphoma cells (HDLM-2line) were cultured in RPMI-1640 supplemented with 20% FBS, 50 U/ml penicillin, 50 µg/ml Streptomycin, 2 mM L-glutamine, and 1 mM sodium pyruvate (all from Gibco) in 37 °C humidified incubator with 5% CO_2_ atmosphere. The cells were sub-cultured every 3 days. For microscopy experiments the cell pellet was re-suspended in 300 µl of PBS (pH ~7.3) and placed on poly-L-lysine-coated 170 µm-thick coverslips (Schott AG, Germany) and left for approx. 30 s to settle down. Subsequently, the coverslips were transferred to 3.7% formaldehyde (Sigma-Aldrich) in PBS for 15 min, washed once with PBS and incubated with 0.5% TX-100 (Sigma-Aldrich) for 15 min. Cells were stained with 50 µg/ml Hoechst 33258 (Sigma Aldrich) for 1 h at 37 °C followed by one wash with PBS. Cells were embedded in ~60 µl freshly prepared mounting media (see Supplementary Table 1 for detailed procedures), placed onto glass slides and sealed using hardening mounting agent Castasil 21 (Holland Dental, The Netherlands).

### White blood cell isolation and lymphocyte staining

Peripheral blood was obtained from healthy donors who signed informed consent according to the institutional approval of University of Manitoba’s Health Research Ethics Board Protocol (#HS14085(H2011:336)). White blood cells were isolated using Ficoll-gradient centrifugation. Blood was mixed with PBS 3.5:1 and added to a Ficoll in 1.5:1 ratio and centrifuged (210 g, 30 min). The buffy-coat was collected, washed once in a PBS and re-suspended in the same RPMI-1640 medium as used for HL cells, but supplemented with 10% FBS. Cells were cultured in 37 °C for 72 h, placed onto poly-L-lysine-coated slides and fixed as described for HL cells. Lamin A/C was labelled via 1 h incubation with 1:250 rabbit antibody (ab26300, Abcam, UK) followed by 1 h incubation with Alexa647 antibody (A-31573, Thermo-Fisher, Canada).

### Mouse spleen sections

Formalin-fixed and paraffin-embedded male mouse spleen 10 µm-thick sections were cut on a microtome, placed on glass slides and incubated at 37 °C overnight. Sections were stored at −20 °C. Paraffin was removed using xylene (2 × 15 min for 10 µm- and 3 × 15 min for 20 µm-thick sections, Sigma-Aldrich) and tissue rehydration proceeded through incubation in solutions of descending concentration of ethanol: 100, 90, 70%, 5 min each. Subsequently, sections were incubated for 5 min in PBS and permeabilised using 0.75% TX-100 in PBS for 30 min at 37 °C. Nuclear DNA was stained through overnight incubation with 50 µg/ml Hoechst 33258 followed by a wash in PBS. 10 µm-thick sections were cleared using mounting media as described in Supplementary Table 1; 20 µm-thick sections were incubated in 25% wt/wt, 40% wt/wt Histodenz, 37 °C, 30 min each, followed by 2 h incubation in H71VE at 37 °C. 170 µm-thick coverslip (Schott AG, Germany) was placed on top of the specimen and an excess of mounting medium was removed by gently pressing with a tissue paper to locate a coverslip as close to the specimen as possible. Sample was sealed using Castasil21. All animals were handled in strict accordance with good animal practice as outlined by the University of Manitoba Research Ethics and Compliance Committee and Animal Care & Veterinary Services, and was approved by said University of Manitoba Ethics and Animal Care Services.

### Mounting media preparations

Mounting media based on solutions of sucrose, D-(-)-fructose, D-sorbitol, and Histodenz were prepared freshly in PBS (pH = 7.3) by rapid stirring and heating up to 37 °C (fructose was incubated in 55 °C) for approx. 1–2 h followed by an addition of Vectashield H-1000 (Vectorlabs, USA). Air bubbles were removed by a short centrifugation of each medium. Detailed concentrations of clearing agents can be found in Supplementary Table 1. pH values of the mounting media were: Vectashield, 7; H71VE, 7.7, Sorb70VE, 6.7; S65VE, 7.3; F80VE, 6.2. Refractive indices of the mounting media were measured at 25 °C and 589 nm wavelength using Abbe refractometer (DR201-95, Kruess, Germany). Supplementary Table 2 contains measured values of the refractive index for various combinations of the mounting media studied in this work. All reagents were purchased from Sigma-Aldrich, Canada.

### 3D-SIM measurements and analysis

Measurements were performed using Zeiss PS.1 Elyra microscopy system equipped with 63× 1.4 NA objective lens (pixel-size in acquired pictures: 79 nm) and IXon 885 EMCCD Camera (Andor, UK) except Supplementary Fig. S5 where OMX v3 Blaze SIM system was used (kindly made available by Micron Advanced Bioimaging Unit). Immersion oils with refractive index between 1.508 and 1.520 (GE Healthcare, UK) were used. All images of tissue sections were acquired with standard 1.518 immersion oil. 100% 405 nm laser power was used for excitation (corresponding to <0.1 kW/cm^2^). 28 µm SIM grating period was used. Each SIM image was reconstructed from 15 images in total, i.e. 3 grid orientations with 5 phases were used for data presented in Figs 1–3. Camera was operated with no additional gain and with 150 ms exposure time. Z-step of 150 nm was used in data presented in Figs 1 and 2 and 125 nm for data in Fig. 3. Image reconstruction was performed using the ZEN software (Zeiss, Germany) and 39 nm pixel-size in reconstructions was specified. Noise filter value (corresponding to Wiener filter) was selected from a range between −2.1 (for raw data with low MCNR, e.g. in Vectashield) to −4.0 (for data with high MCNR >8, e.g. in H71VE). Most commonly used noise filter settings for good quality data amounted to approximately −2.9. Theoretical point spread function (PSF) was used for all reconstructions. MCNR and average frequency spectra in raw 3D-SIM images were generated using *SIMcheck* software^19^ operating under ImageJ/FiJi platform^39^. Frequency spectra from many individual 3D-SIM measurements were averaged using ImageJ and their radial profiles (Fig. 3G) were obtained using *Radial Profile Angle* ImageJ plugin integrating the signal over 360° with respect to the frequency spectrum center. To obtain MCNR profiles across imaging depths MCNR 3D maps were first tresholded, i.e. MCNR values below 4 were set to “not a number” (NaN) value. Next, ImageJ function “Plot Z-axis profile” was used to obtain average MCNR Z-profiles. Tissue section thickness differed slightly from measurement to measurement hence they were aligned to match each other’s maximal value at the imaging plane close to the coverslip (MCNR peak equal to 1, Supplementary Fig. S7B). More than 3 individual curves for independent 3D-SIM measurements were averaged and presented in Supplementary Fig. S7. Intensity histogram in final 3D-SIM reconstructions was stretched between a peak corresponding to the values of low intensity background and the maximal intensity value in the 3D image using ZEN software unless otherwise stated. Statistical significance was assessed using t-test. P-value <0.001, 0.001–0.01, and 0.01–0.05 is indicated with three, two, and one asterisk.

### Assessment of photobleaching

Photobleaching of Hoechst-, Alexa488-, Alexa555-, Cy3-, Alexa594- labelled HL cells was studied using Zeiss Elyra PS.1 microscope operating in PALM mode. Movies between 200–700 frames (30 or 50 ms each) were acquired under exposure to exciting light (same settings for each fluorophore in different media). Grey values <2000 units corresponding to camera background were discarded and total fluorescence intensity above the background was measured using Time Series Analyzer plugin (ImageJ). Four or more measurements were averaged and normalised to the first frame for each curve (Supplementary Figs S9 and S10).

### SMLM & correlative SMLM-SIM

SMLM measurements were performed using a custom-built microscope at the Department of Cell Biophysics, Jagiellonian University, based on Olympus IX-73 body. Alexa647 was excited using a 120 mW 647 nm laser (Calypso 06-01, Cobolt, Sweden). For SMLM an alternative beam path has been introduced containing ~4:1 beam expander (Thorlabs, Sweden). Subsequently, the exciting light was introduced to a microscope back-port and reflected from a beam-splitter (90100bs, Chroma, Germany) to 100× 1.49NA objective lens (Olympus, Japan). The resulting illumination area in a sample plane had a diameter of approx. 20 µm. Fluorescence signal passed through a 655LP emission filter (et655lp, Chroma, Germany) and was focused by a 1′′ achromatic doublet lens (f = 300 mm, AC254-300-A-ML, Thorlabs, Sweden) on to a 512 × 512 px^2^EMCCD camera (IXon+, Andor, UK). The resulting camera pixel size corresponded to 96 nm in the sample plane. SMLM measurements of Alexa647–labelled Lamin B1 samples embedded in various mounting media were performed with ~2 kW/cm^2^ 647 nm laser intensity, 3000 × 25 ms frames. SMLM reconstructions, photon counts, and localisation precision were measured using ThunderSTORM^40^. 1.5x STD signal detection threshold was set and signals appearing in subsequent frames were combined using 1x average localisation uncertainty threshold. The same software settings were used to detect fluorescent beads and measure their integrated relative intensity (Supplementary Fig. S11). For correlative super-resolution imaging of lymphocytes stained for Lamin A/C (Alexa647, SMLM) and DNA (Hoechst 33258, 3D-SIM) they were embedded in S65VE medium and imaged on Zeiss Elyra PS.1 system equipped with 63× 1.4NA oil-immersed objective lens. Excitation lens was used in u_HP mode and 100% 642 nm laser power was used to induce Alexa647 fluorescence signal fluctuation (approx. intensity of 0.2 kW/cm^2^ in sample plane). Signal was detected using EMCCD camera (Andor, UK) with 100 ms exposure time and 10 V gain. 5000 frames in total were acquired. All SRRF-SMLM images (Supplementary Fig. S16) were reconstructed using NanoJ-SRRF ImageJ plugin with the following settings^32^: Ring Radius: 1.6, Radiality Magnification: 5, Axes Ring: 4, Temporal Radiality Maximum, TRAC order: 2, others were set to default. Subsequently the same cells were imaged in 3D-SIM mode using 405 nm laser as described in Materials and Methods for HL cells.

## Electronic supplementary material

Supplementary Information

## Data Availability

The datasets generated during and analysed during the current study are available here: http://helios.wbbib.uj.edu.pl/szczurek-et-al.-2018-scientific-reports.
