# Supplementary material for: Aqueous mounting media increasing tissue translucence improve image quality in Structured Illumination Microscopy of thick biological specimen
Source: Sci Rep. 2018 Sep 18;8:13971. doi: 10.1038/s41598-018-32191-x (PMC6143540; doi:10.1038/s41598-018-32191-x)
Supplement: Supplementary file 1 — Supplementary Information [file 41598_2018_32191_MOESM1_ESM.pdf]

SUPPLEMENTARY INFORMATION

Aqueous mounting media increasing tissue translucence improve image quality in Structured Illumination Microscopy of thick biological specimen

Aleksander Szczurek[1,2], Fabio Contu[1,3], Agnieszka Hoang[2], Jurek Dobrucki[2], Sabine Mai[1]*

[1] University of Manitoba, Cancer Care Manitoba, Winnipeg, 675 McDermot Ave, R3E 0V9, Canada;

[2] Department of Cell Biophysics, Faculty of Biochemistry, Biophysics and Biotechnology, Jagiellonian University, Krakow, Gronostajowa 7, 30-387, Poland;

[3] University of Cagliari, Unit of Biology and Genetics, Department of Biomedical Sciences, S. P. Monserrato, Sestu Km 0.700,09042, Italy;

*Corresponding author; E-mail: sabine.mai@umanitoba.ca; Phone: +1 (204) 787-2135

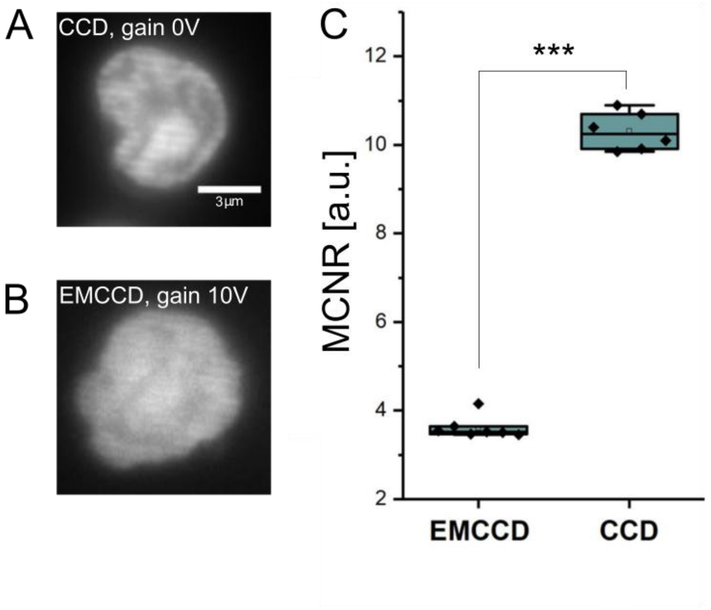

Supplementary Figure S1. **Quantification of SIM modulation contrast to noise ratio (MCNR) value in normal white blood cells (granulocytes and lymphocytes) stained with DAPI DNA-binding probe.** Two different camera settings were used for the sake of demonstration. **A**) An example of a raw frame from a SIM acquisition using a CCD camera with no additional signal amplification. Periodic fluorescence signal induced by structured illumination pattern is visible as stripes oriented horizontally across the cell nucleus. Exciting laser power is set to 100%. **B**) Raw frame from a SIM experiment using a microscope camera in an EMCCD mode (a lower laser intensity and an additional signal amplification). Exciting laser power was decreased by 50% here. At these settings the pattern induced by structured illumination is quite faint. This is reflected in very low values of MCNR presented in (**C**). Total dynamic range for raw 3D-SIM acquisitions (ratio between maximal and minimal intensity value) amounts to: 65532/1968=33.29 for EMCCD mode, and 28056/2000=14.03 for CCD mode. Although the dynamic range for EMCCD camera settings is over 2 times greater than for CCD camera settings, the MCNR values for the latter are significantly higher (see C). We attribute the poor modulation contrast outcomes for EMCCD camera mode to amplification of the faint out-of-focus signals obscuring the illumination pattern in objective's focus. MCNR values >6 typically correspond to acceptable quality of raw SIM data. Camera exposure time was identical in both experiments. More than 6 cells were analysed for each condition. Box outlines represent the values of 25 and 75 percentile as well as the median in between. Error bars correspond to standard deviation.

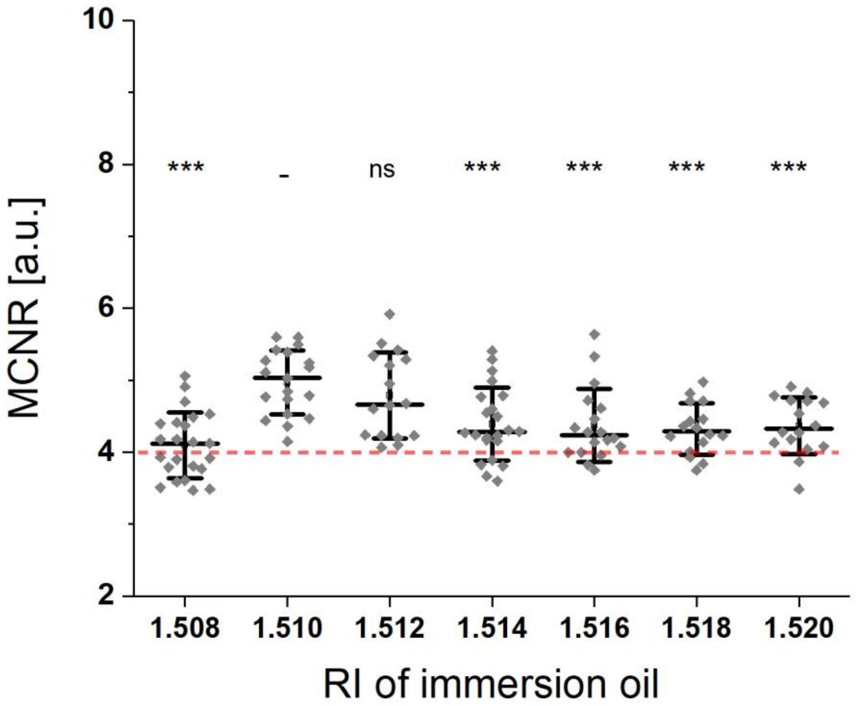

Supplementary Figure S2. Optimisation of refractive index of the immersion oil, and raw 3D-SIM data quality assessment for DAPI-stained DNA of Hodgkin's lymphoma cells embedded in Vectashiled. Each data point corresponds to a single cell 3D-SIM measurement. The highest MCNR value was achieved for immersion oil with RI=1.510 as the best 3D-SIM results were obtained for this immersion oil. Comparable results were obtained with Hoechst 33258 DNA stain (**Fig. 1A**). p-values were calculated using t-test with respect to the results achieved for 1.510 oil.

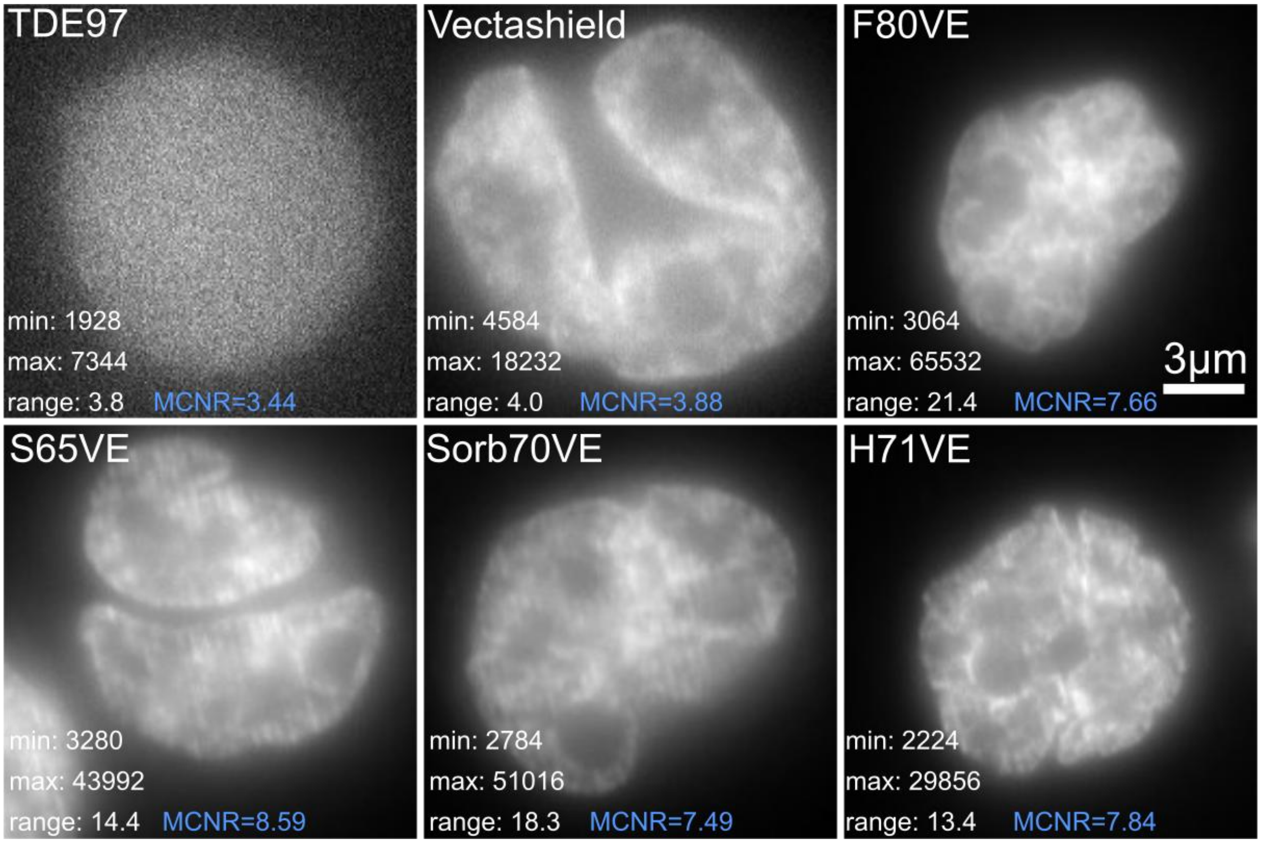

Supplementary Figure S3. Examples of raw SIM data-frames for Hoechst 33258-stained Hodgkin's lymphoma cells embedded in various mounting media. Imaging depth was 5 - 10 µm, immersion oil used for all images had RI of 1.518. Vertical stripes are induced by SIM illumination pattern and are clearly visible in cell samples where new SIM-dedicated mounting media were applied. All images were acquired with the same settings, although the final brightness/contrast was adjusted linearly for presentation. An exception was the image of a cell embedded in TDE97; here a higher camera gain was used to offset low signal intensity. Minimal, maximal values together with image dynamic range value and respective MCNR are given for each image (left-bottom). No clear correlation is discernible between the dynamic range (expressed as max/min ratio) and MCNR. For instance the dynamic range for F80VE medium is approx. 2x greater than the one for H71VE medium even though both media scored strikingly similar MCNR values. The images obtained in Vectashield have typically approx. 2x lower Hoechst 33258 max intensity. See **Supplementary Table 3** for more information on entire datasets. Varying dynamic range values may stem from the fact that in some media the initial signal intensity might be higher but susceptible to photobleaching to a different extent. For more details on initial fluorescence intensity in various media see **Supplementary Figure S11**. See **Supplementary Figure S5** for quantitative comparison of 3D-SIM measurements acquired with varying exciting light intensity and camera integration time settings for a comparable dynamic range between measurements and conditions. See **Figures 1, 2** for more details and quantitative assessment of periodic artefacts induced by the illumination pattern in fluorescence images. Scale bar applies to all images in this figure and corresponds to 3 µm.

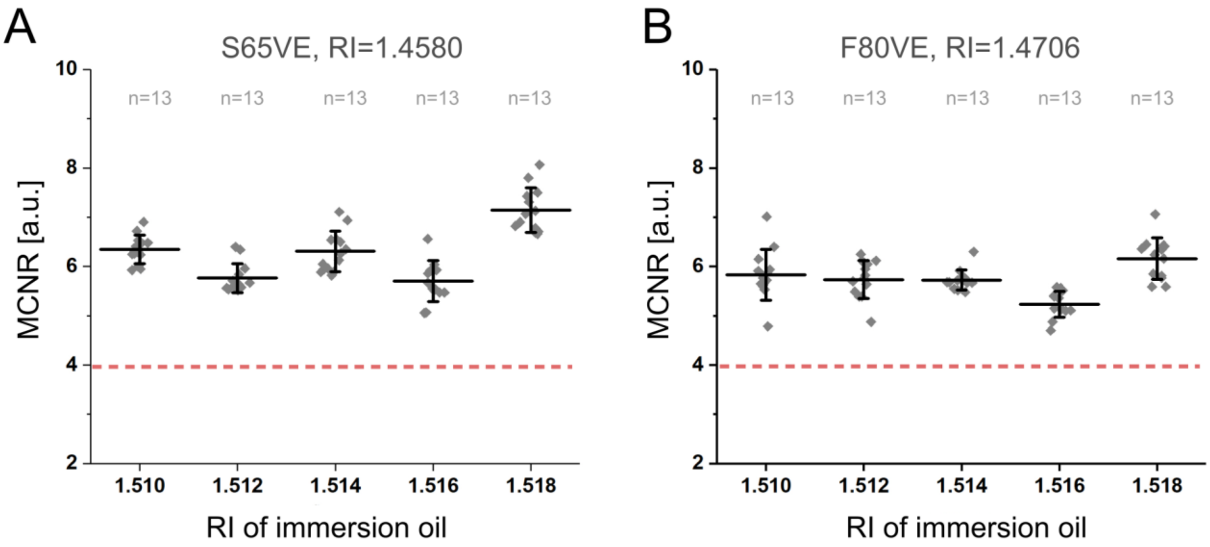

Supplementary Figure S4. Quantitative comparison of raw SIM data quality. MCNR calculated for Hodgkin's lymphoma cells stained with Hoechst 33258, and embedded in S65VE (**A**) and F80VE (**B**). The red dashed line indicates MCNR threshold value equal to 4 below which the data do not pass the evaluation and are not acceptable for meaningful SIM. Each datapoint represents a single cell measurement. Mean and standard deviation values indicated in black. All measurements were statistically different from respective measurements carried out in Vectashield (see **Fig. 1A**), i.e. p-value in t-test <0.001.

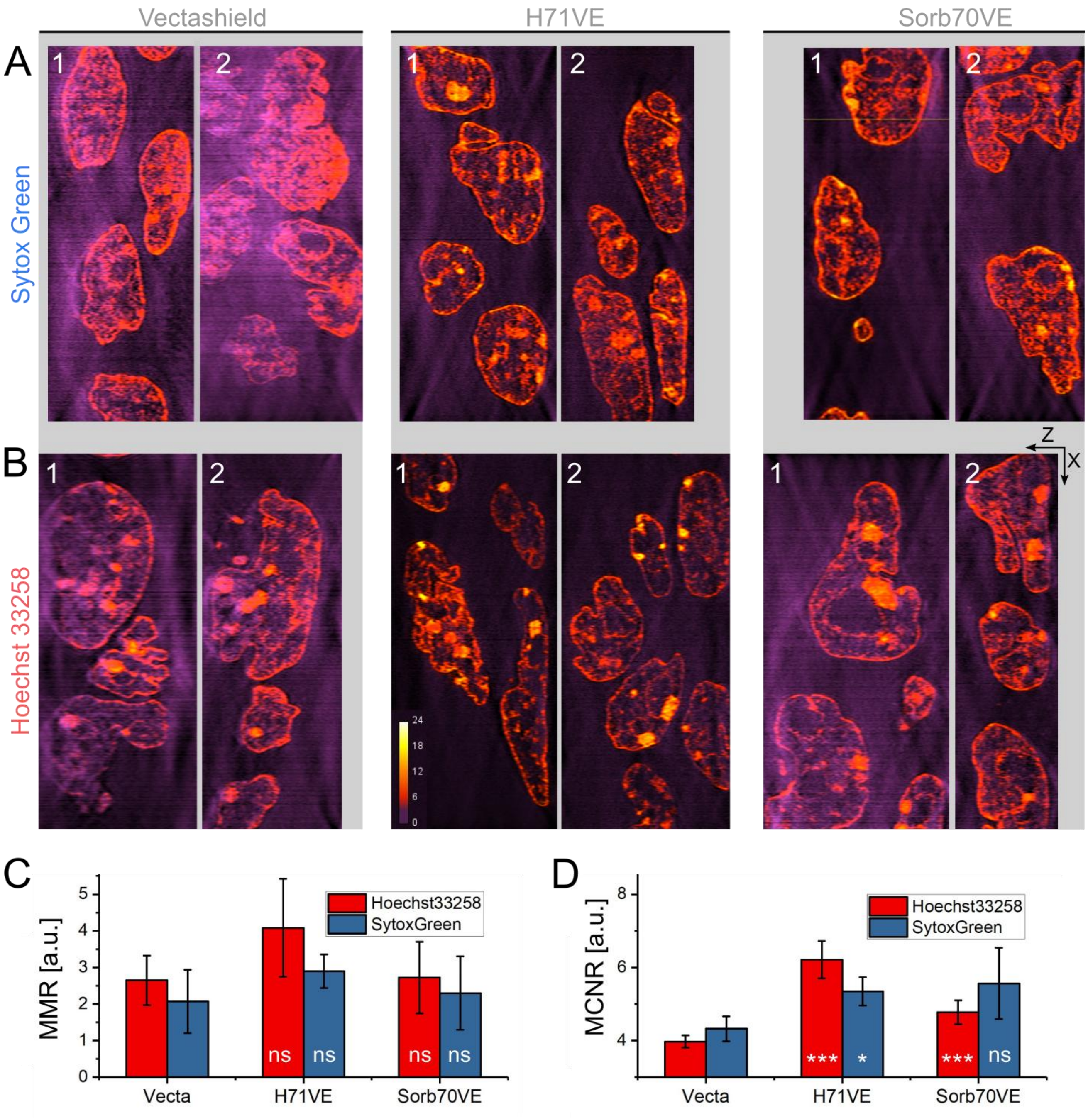

Supplementary Figure S5. 3D-SIM data evaluation of thick mouse embryonic stem cells stained for the DNA using measurement-specific system settings. The cells were stained with Sytox Green (**A**) or Hoechst 33258 (**B**) and embedded in Vectashield (left), H71VE (middle) and Sorb70VE (right). Two examples of orthogonal sections (1 and 2) are presented for each condition. As opposed to other experiments presented in this work, fixed exciting light intensity and camera integration time settings were not used in this experiment. Instead they were adjusted so that the pixel intensities in raw 3D-SIM movies reach the maximal values. This provides most comparable dynamic range between conditions and measurements. 3D-SIM images with overlaid MCNR map presented in A and B (two examples of orthogonal views for each condition) were reconstructed individually using OMX DeltaVision softWoRx software with optimal input Wiener filter value estimated beforehand with SIMcheck based on raw data (Ball et al., 2015). Mean Wiener filter values for the conditions were: Sytox Green: 0.009+/-0.001

(Vectashield); 0.0062+/-0.0009 (H71VE); 0.0066+/-0.0017 (Sorb70VE). Hoechst 33258: 0.0110+/-0.0091 (Vectashield); 0.0045+/-0.0008 (H71VE); 0.0075+/-0.0011 (Sorb70VE). Right-most imaging planes in A and B correspond to planes closest to the microscope objective. Mean max-to-min intensity ratios (MMR) and mean modulation contrast (MCNR) for each imaging condition are presented as bar plots (**C** and **D**, respectively). 3 to 6 mES cell groups were measured per each condition. As a result of this evaluation, H71VE, the medium with RI matching the system optics best, was ranked highest among the conditions tested. Note that MMR values for all the mounting media do not significantly differ.

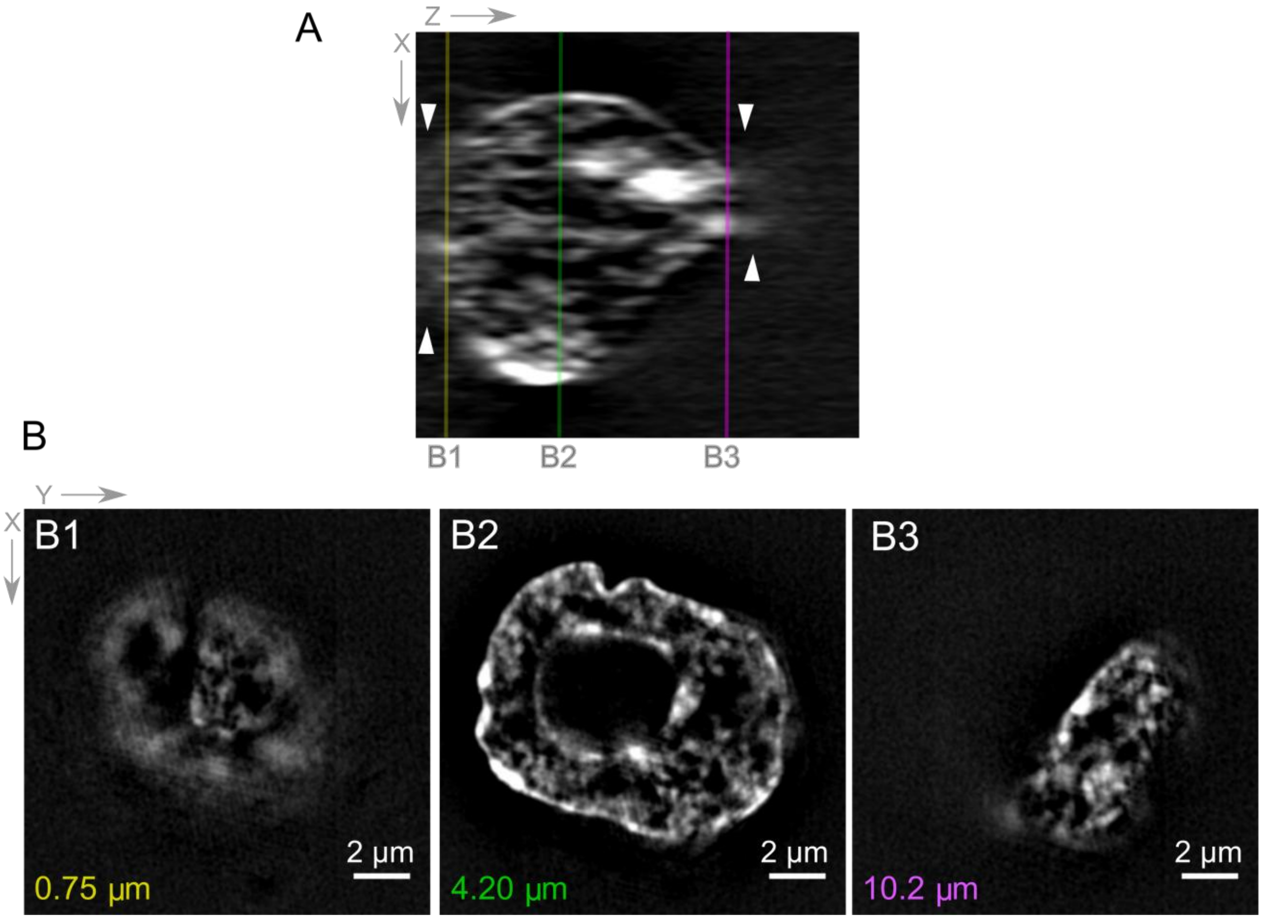

Supplementary Figure S6. An example of 3D-SIM reconstruction of a Hodgkin's lymphoma cell stained with Hoechst 33258 and embedded in Vectashield. Analogous data for H71VE medium are presented in **Figure 2**. **A**) Orthogonal view (XZ) of a 3D-SIM reconstruction of a lymphoma cell with marked horizontal cross-sections (lines, B1-B3). Arrowheads indicate signal smear above and below the cell nucleus. This artefact is usually absent when imaging is performed in S65VE, Sorb70VE, and H71VE. **B**) Horizontal views (XY) at different imaging depths: B1) 0.75 μm, close to the coverslip; B2) 4.2 μm; and B3) 10.2 μm, far from the coverslip. Images at every depth bear characteristic grid-like artefacts: a hallmark of poor illumination pattern quality at imaged plane (see **Supplementary Fig. S3** for more details).

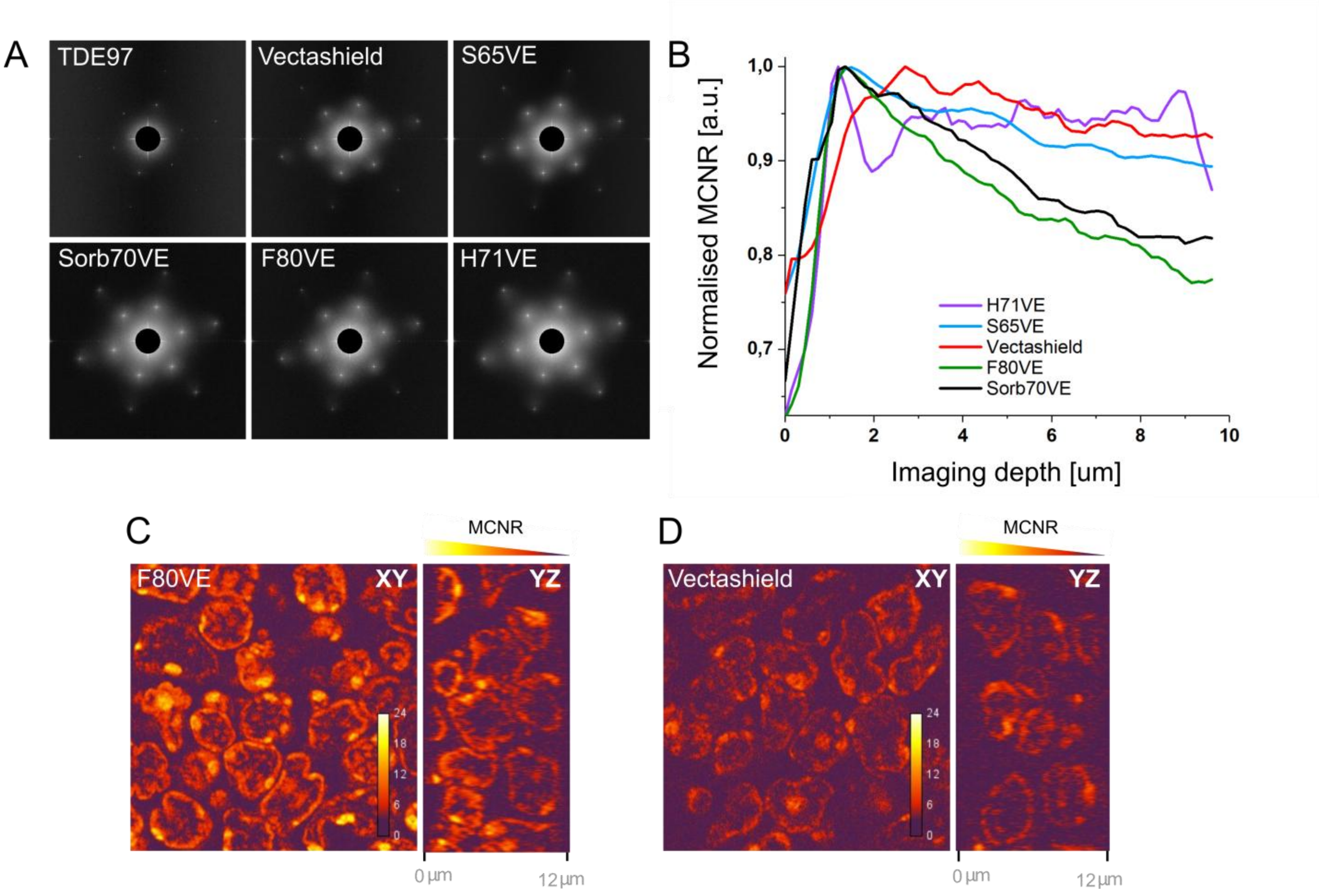

Supplementary Figure S7. 3D-SIM imaging of mouse spleen tissue sections stained with Hoechst 33258 and cleared using various mounting media. A) Examples of average FT spectra from >3 measurements. These spectra are plotted as radial curves presented in **Figure 3G**. TDE97 (97% v/v 2,2'-thiodiethanol in water) mounting medium was previously proposed as a refractive index matching mounting medium for high-resolution microscopy with reduced spherical aberration (Staudt et al. 2007). 2,2'-thiodiethanol is known to quench fluorescence of many fluorophores. **B**) Normalised SIM modulation contrast to noise ratio (MCNR) across the entire imaging depth demonstrates depth-dependent decrease of MCNR for some of the SIM mounting media tested. MCNR value obtained for spleen sections in TDE97 was too low to assess its profile across the depth of imaging. For absolute MCNR values see **Figure 3F**. **C**) An example of the MCNR map (XY) with an associated orthogonal view (YZ) for F80VE-embedded section revealing a substantial decrease in MCNR at ~10µm imaging depth. Respective MCNR values are included in **Fig. 3**. **D**) MCNR map (XY) with an example of an orthogonal view (YZ) for Vectashield. Note that MCNR value for Vectashield, although low, is rather constant across the imaging depth.

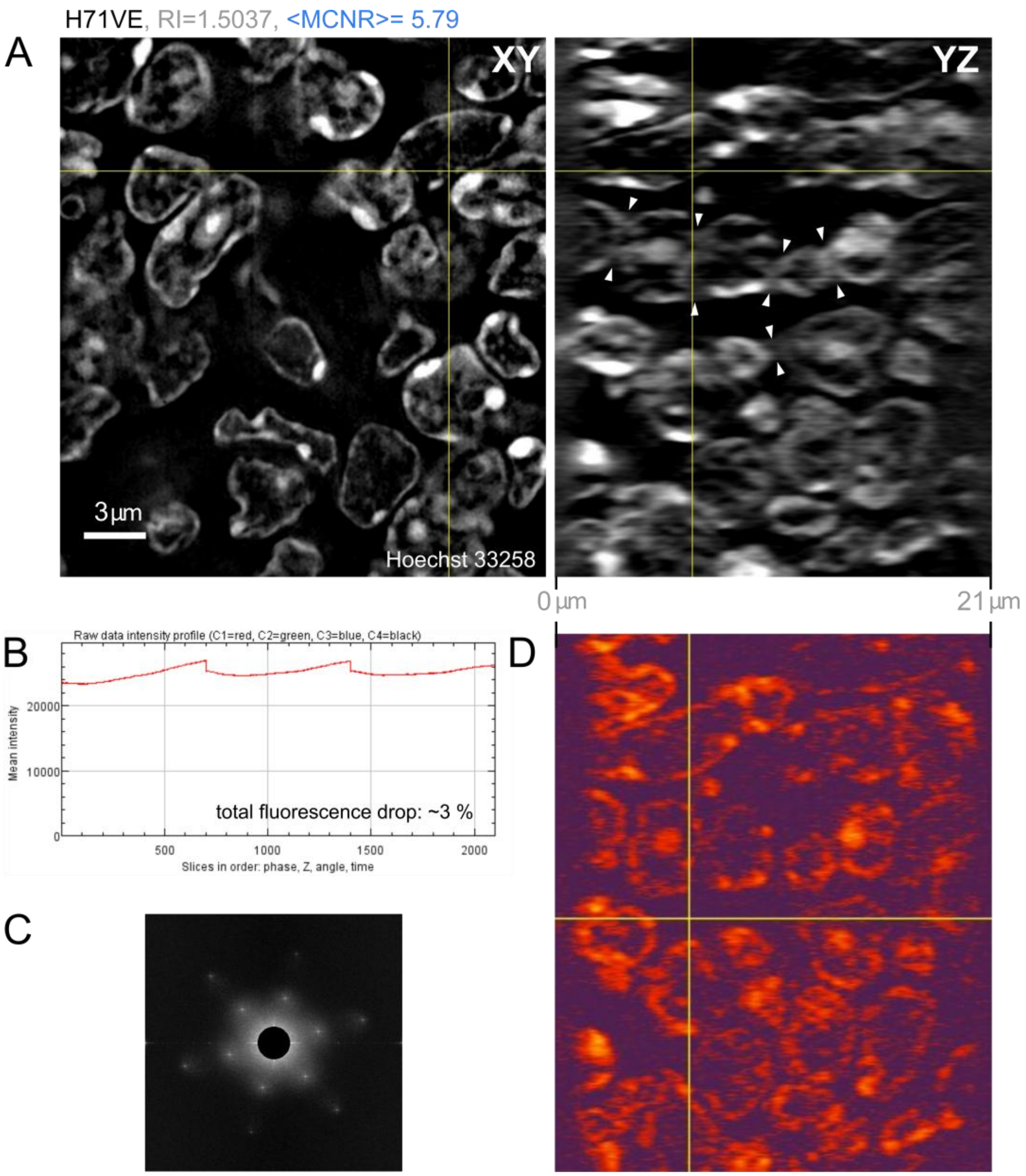

Supplementary Figure S8. Evaluation of raw 3D-SIM data of 20 µm-thick mouse spleen sections stained with Hoechst 33258 and subsequently cleared with H71VE. A) 3D-SIM image reconstruction; horizontal view (left) with an orthogonal view (right) through a 3D-SIM image. Arrowheads indicate areas of the image where the compromised axial resolution is clearly visible (stacked nuclei are merged). Though the resolution improvement in SIM image is evident, the quality of image is impaired throughout entire imaging volume as compared to the results for 10 µm-thick sections (see **Supplementary Fig. S7**). **B**) Fluorescence intensity variation plot for the image (3 grid phases, 5 orientations) reveals only minor photobleaching of Hoechst 33258 during the experiment. **C**) Frequency spectrum of the 3D image in A. **D**) Orthogonal view across MCNR map for raw 3D data of SIM data from A. In order to compare this outcome with the one obtained for 3D-SIM of 10 µm-thick tissue sections see **Supplementary Fig. S7C** and **Fig. 3**. B-D were obtained using *SIMcheck* (Ball et al. 2015).

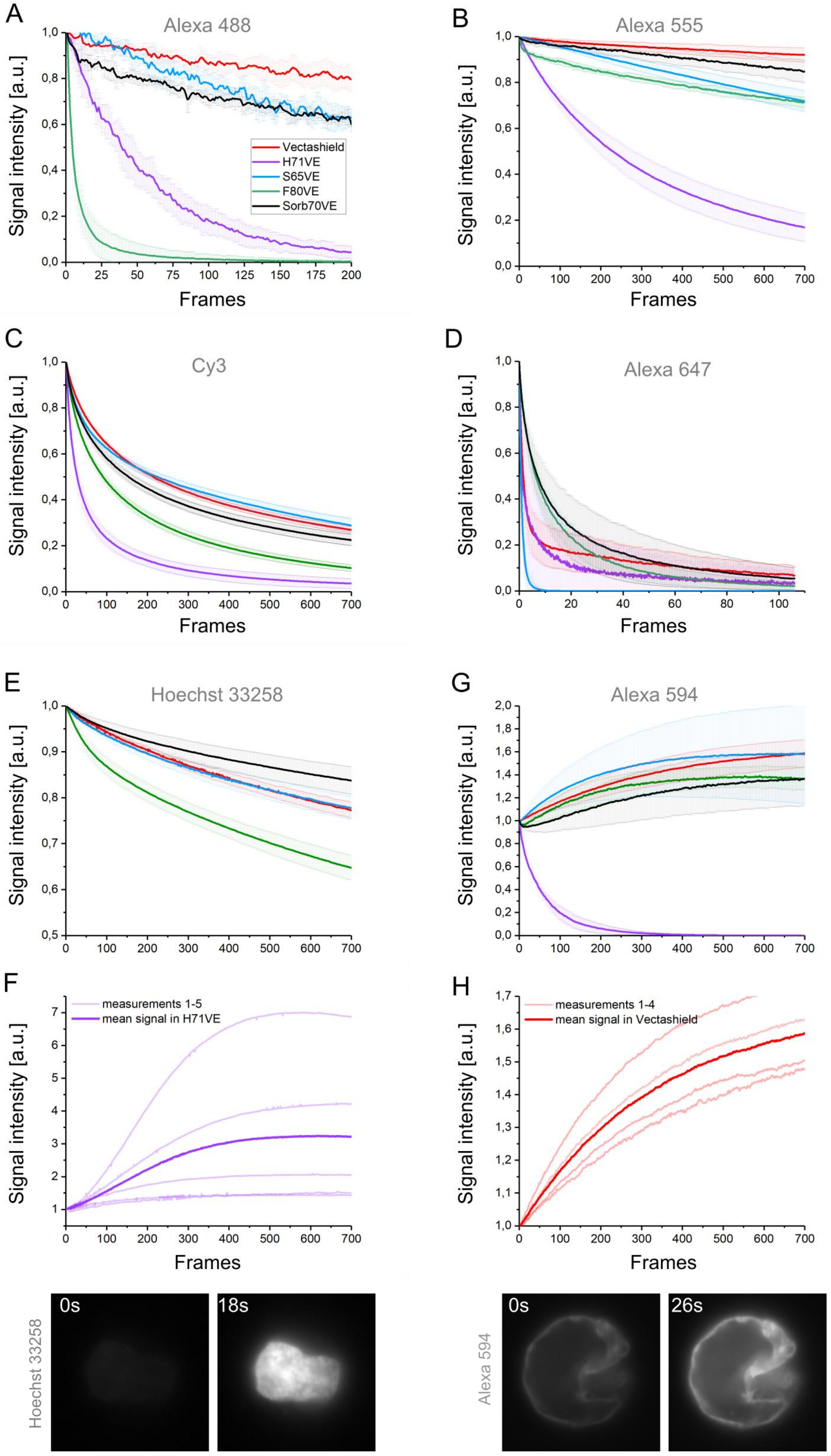
A
Alexa 488
Vectashield
H71VE
S65VE
F80VE
Sorb70VE
Signal intensity [a.u.]
Frames
B
Alexa 555
C
Cy3
D
Alexa 647
E
Hoechst 33258
G
Alexa 594
F
measurements 1-5
mean signal in H71VE
H
measurements 1-4
mean signal in Vectashield
0s
18s
Hoechst 33258
26s
Alexa 594

Supplementary Figure S9. Photostability of commonly used fluorescent probes for multicolour 3D-SIM embedded in various mounting media for 3D-SIM of thick objects. Nuclear lamina in HL cells was stained by immunofluorescence, using secondary antibodies conjugated with various fluorescent probes, and DNA, using Hoechst 33258. The cells were exposed to exciting light and the total fluorescence signal intensity was measured over time. Signal intensity decays corresponding to Alexa 488 (exc. 488 nm), Alexa 555, Cy3 (exc. 561 nm) and Alexa 647 (exc. 647 nm) are presented in **A**, **B, C** and **D**, respectively. Fast Alexa 647 fluroescence signal decrease can be attributed not only to photobleaching but also to light-induced long-lived non-fluroescent reduced state (**Fig. 4**). **E-F** demonstrate fluorescence signal intensity measured for the cells with nuclear DNA labelled using Hoechst 33258. For clarity of data presentation the fluorescence signal for Hoechst 33258 in H71VE medium is presented in a separate panel in F where single measurements and the averaged curves are presented together. Note a striking variability in the signal intensity monitored over time of exposure to exciting light for 5 independent fluorescence measurements (1-5). Interestingly, the signal intensity of Hoechst 33258 increases on average by 3-fold during the time of the experiment (F). Below: example of cell images taken in the beginning and at the end of the experiment demonstrate fluorescence signal increase in the presence of H71VE medium upon exposure to exciting light (405 nm). **G-H** presents similar behaviour of Alexa 594 (exc. 561) with an example of the fluorescence increase in the presence of Vectashield (**H**). Images of the cells immuno-labelled for nuclear lamina using Alexa 594 recorder in the beginning and at the end of the experiment. Note that essentially no Alexa 594 signal decrease occurs in all the media except H71VE. The same curve colour-coding is used in panels A-H. Since the composition of Vectashield has not been disclosed by the supplier, we are unable to reliably postulate a chemical nature of signal increases presented in F and H. The effects visible in G-H could be attributed to slow dye photobleaching leading to a decrease of fluorescence self-quenching.

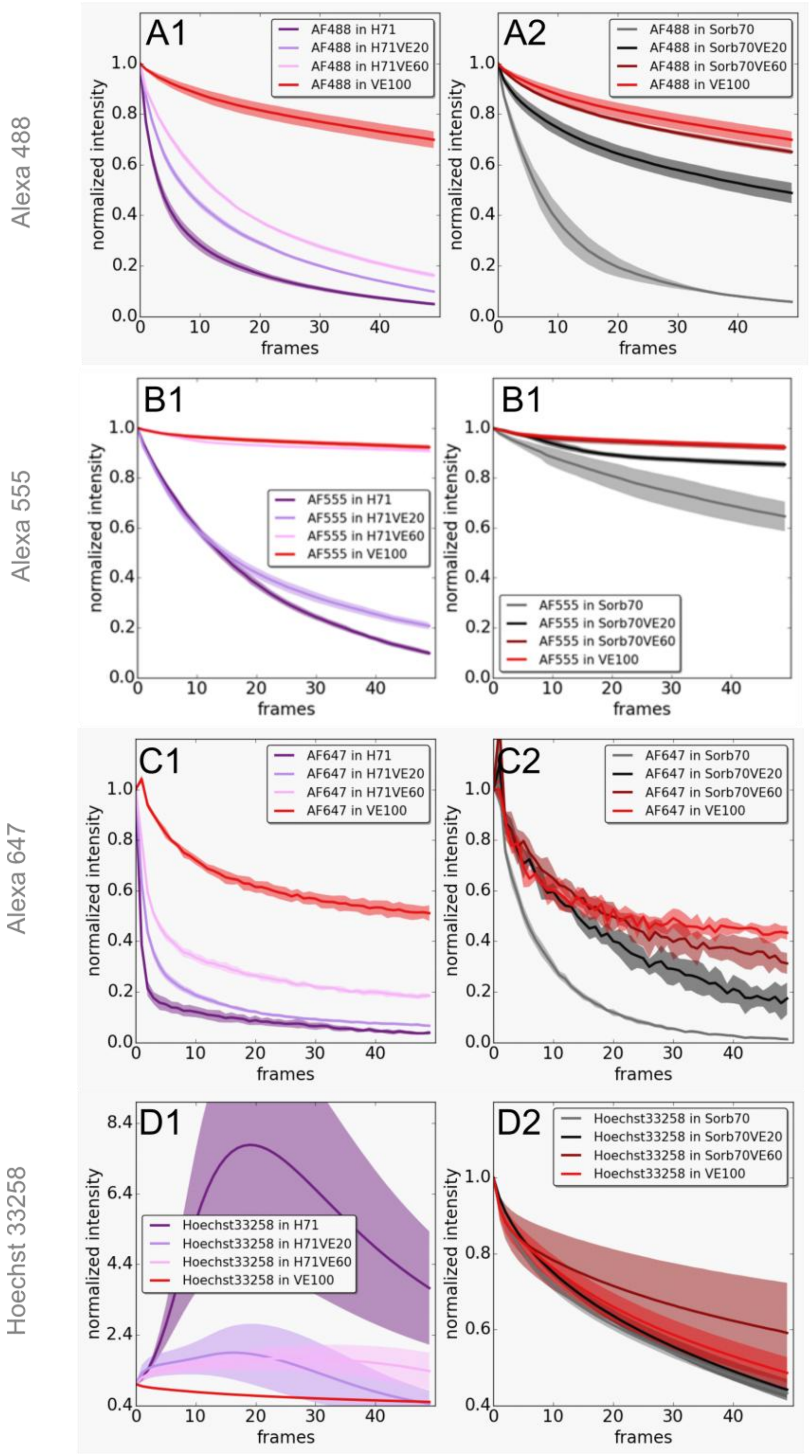

Supplementary Figure S10. **Photobleaching kinetics with varying Vectashield concentration in the clearing media.** 100% (VE100), 60% (VE60), 20% (VE) and 0% Vectashield in Histodenz- and Sorbitol-containing media were tested for Alexa488 (**A1-2**), Alexa555 (**B1-2**), Alexa647 (**C1-2**), and Hoechst 33258 (**D**). Note that in the case of media containing 60% Vectashield protection from photobleaching may reach comparable performance to pure Vectashield. Moreover, the concentration of clearing agents, e.g. Histodenz, can be further increased from 71% wt/wt to e.g. 75% further elevating RI of the mounting media. E.g. in our hands Alexa 555 in H75VE60 medium performs well in SIM.

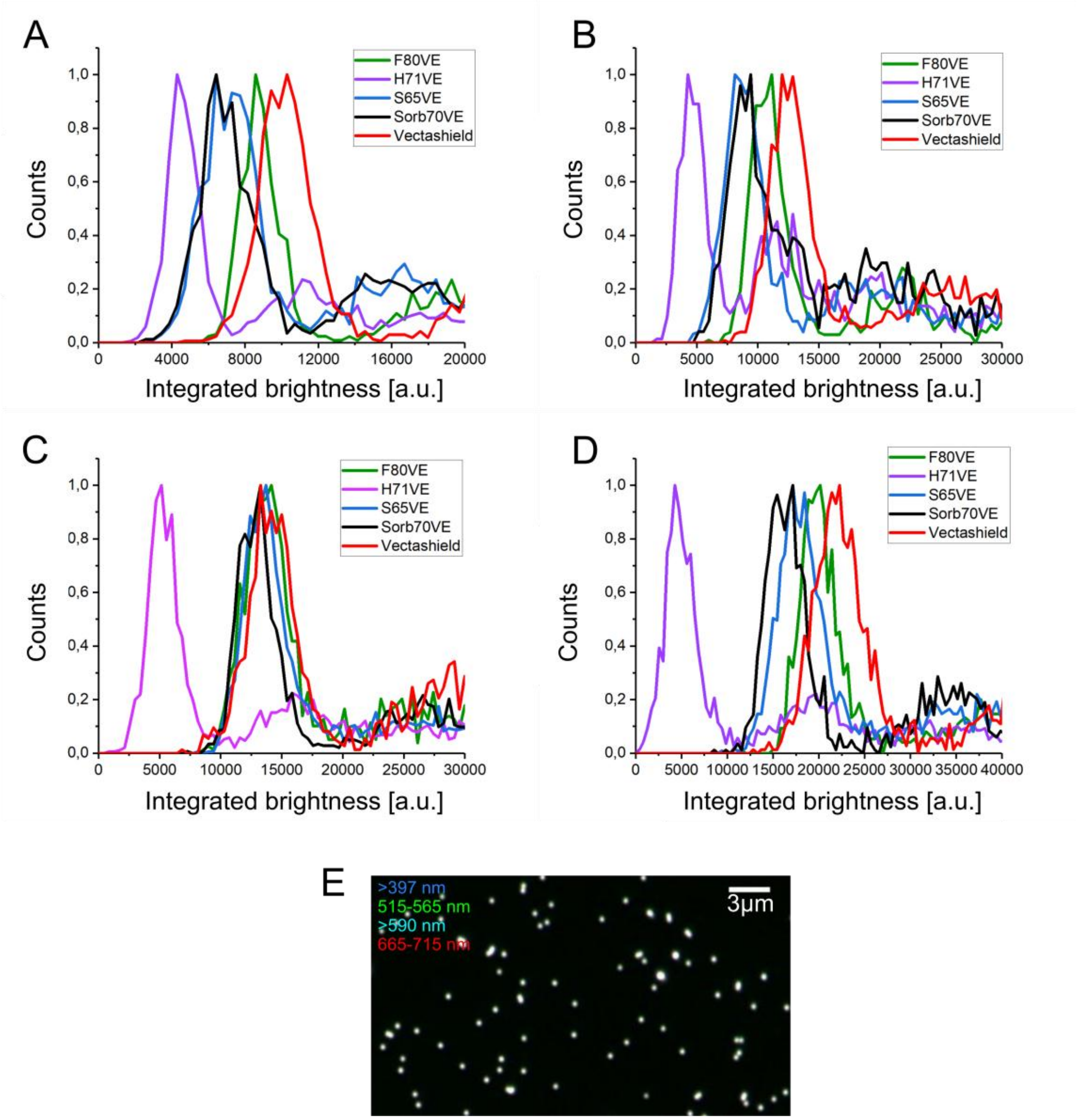

Supplementary Figure S11. **Initial fluorescence intensity comparison of 4-colour 200 nm-microspheres dried on microscope coverslip and submerged in various mounting media**. Total relative fluorescence signal intensity of each bead was measured using SMLM software (Ovesný et al. 2014) and plotted as a histogram (>1000 beads each). Large peaks correspond to single beads whereas smaller peaks at higher intensities correspond to multiple beads too close to be separated. **A**) Fluorescence intensity histogram for DAPI channel (397LP emission filter). **B**) Fluorescence intensity histogram for FITC channel (515 - 565BP emission filter. **C**) Fluorescence intensity histogram for Cy3 channel (590LP emission fitler). **D**) Fluorescence intensity histogram for Cy5 channel (665 – 715 nm emission filter). **E**) An example of a raw 4-colour image of microspheres submerged in F80VE medium. Note that bead intensity in all 4 emission channels is significantly decreased while beads are submerged in H71VE. In turn the fluorescence intensity in other media is comparable or slightly lower than in Vectashield. Similar effects can be expected for standard fluorescent probes (such as Alexa dyes) whose fluorescence stability was studied in **Supplementary Figures S9 and S10**.

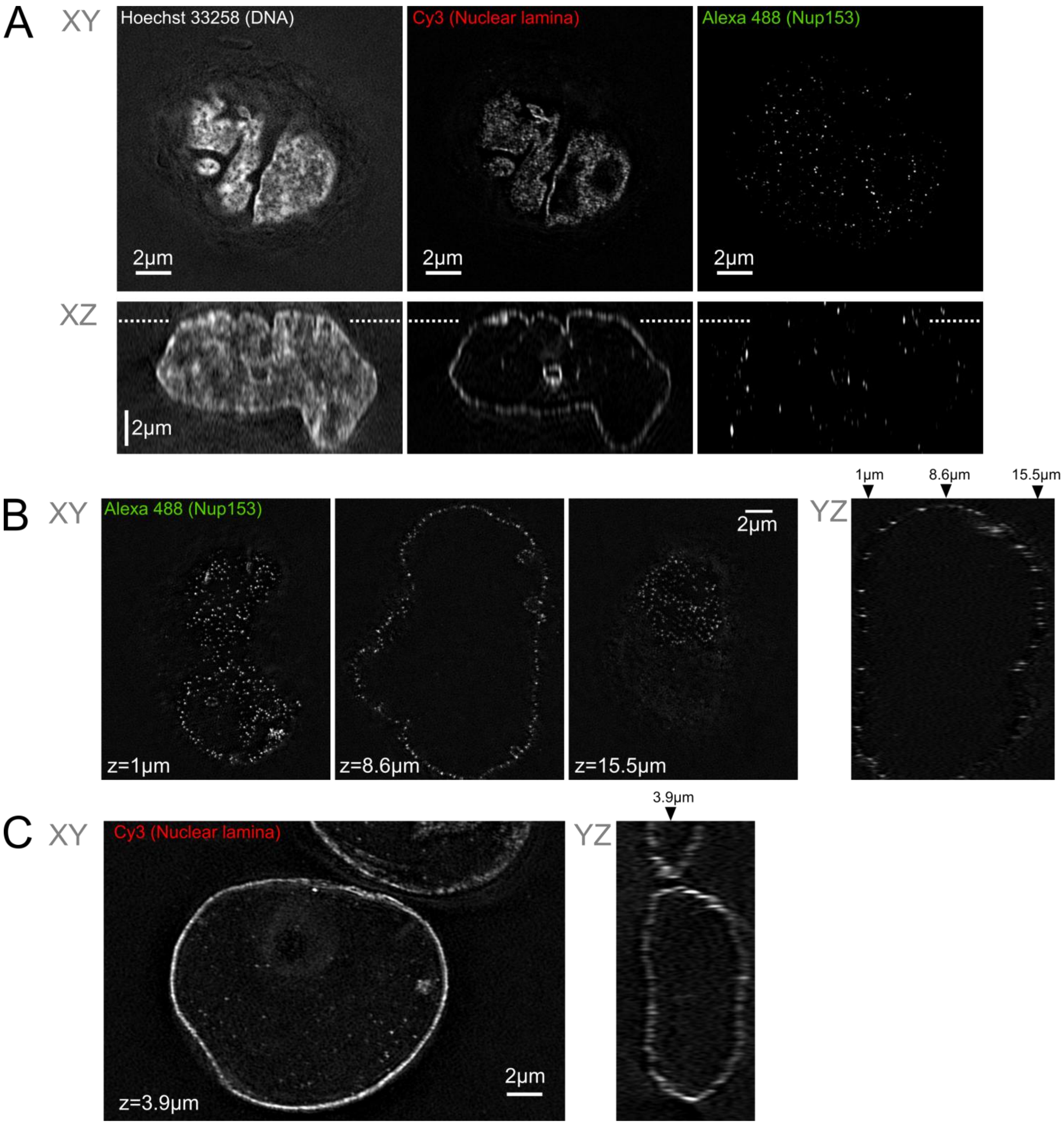

Supplementary Figure S12. Demonstration of multicolour 3D-SIM on immunofluorescently labelled Hodgkin's cells embedded in mounting media studied in this work. A - B) Two examples of HL cells immunolabelled against Lamin A/C and Nup153 nucleoporin using Cy3- and Alexa488- conjugated antibodies. Cells were embedded in S65VE. **C**) Example of HL cell immunolabelled against Lamin A/C using Cy3 and embedded in H71VE medium. Dashed white lines and black arrowheads included in orthogonal views indicate imaging (XY) planes presented on a side.

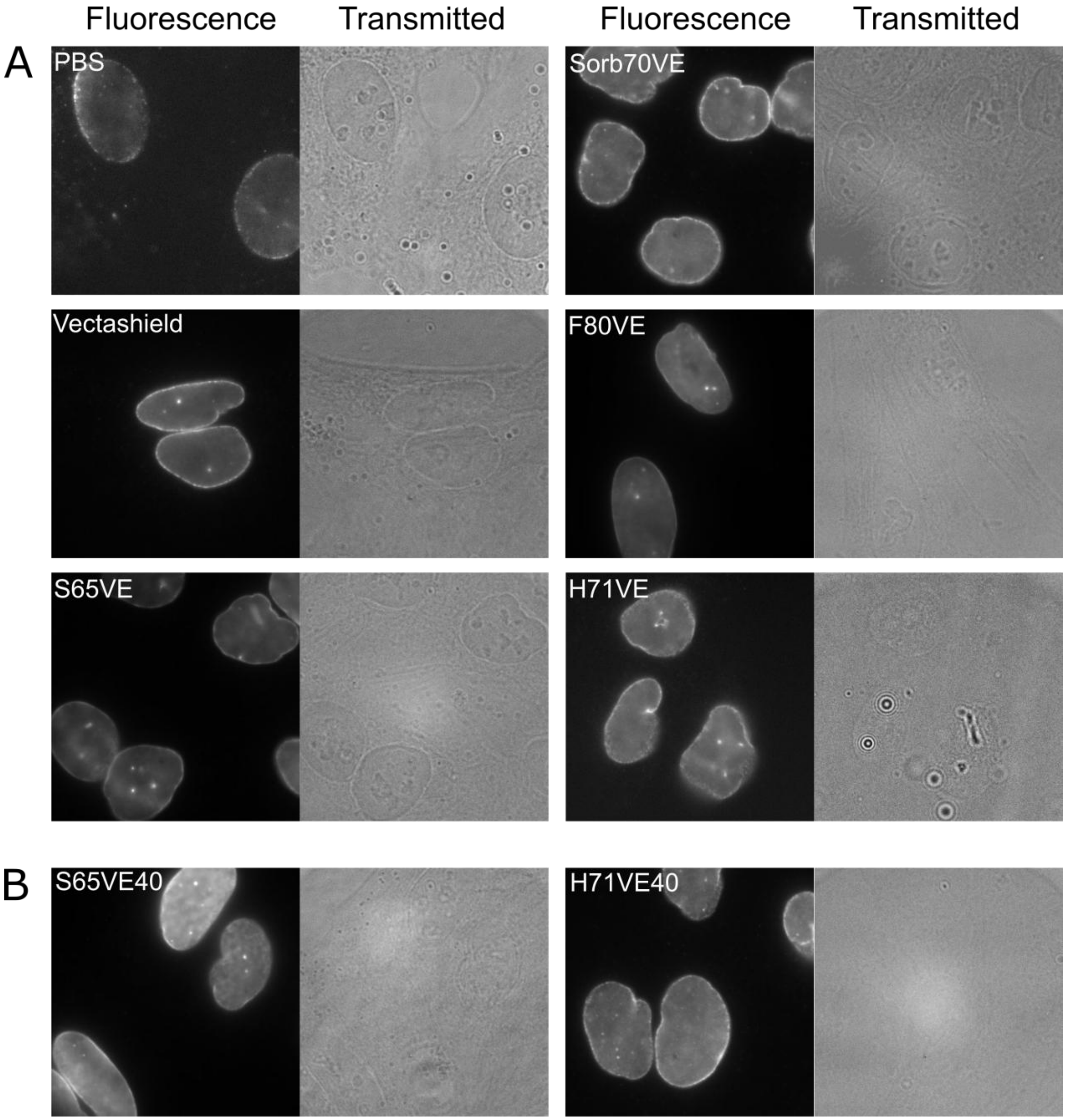

Supplementary Figure S13. Transmitted light images reflect the effectiveness of optical clearing. A549 adherent cells were immunolabelled for Lamin B1 with Alexa 647 and embedded in various media. Fluorescence images (left) together with corresponding transmitted light (TL) images (right) are provided. **A**) Cells submerged in PBS or Vectashield compared with the cells cleared and embedded with SIM-dedicated mounting media described in the first part of this research article, demonstrating loss of contrast when discontinuities of RI are minimised. **B**) TL images for samples embedded in other mounting media containing an increased vol/vol concentration of Vectashield (40%) whose utility in SMLM was evaluated additionally in **Figure 4**. Field of view 50 x 50 μm^2. Cell details in TL images are visible due to light scattering arising from differences in refractive index within the sample. Cell samples with homogenous refractive index, i.e. successfully optically cleared, are barely visible in TL images (compare TL images obtained in SIM-dedicated mounting media to the one with PBS embedding).

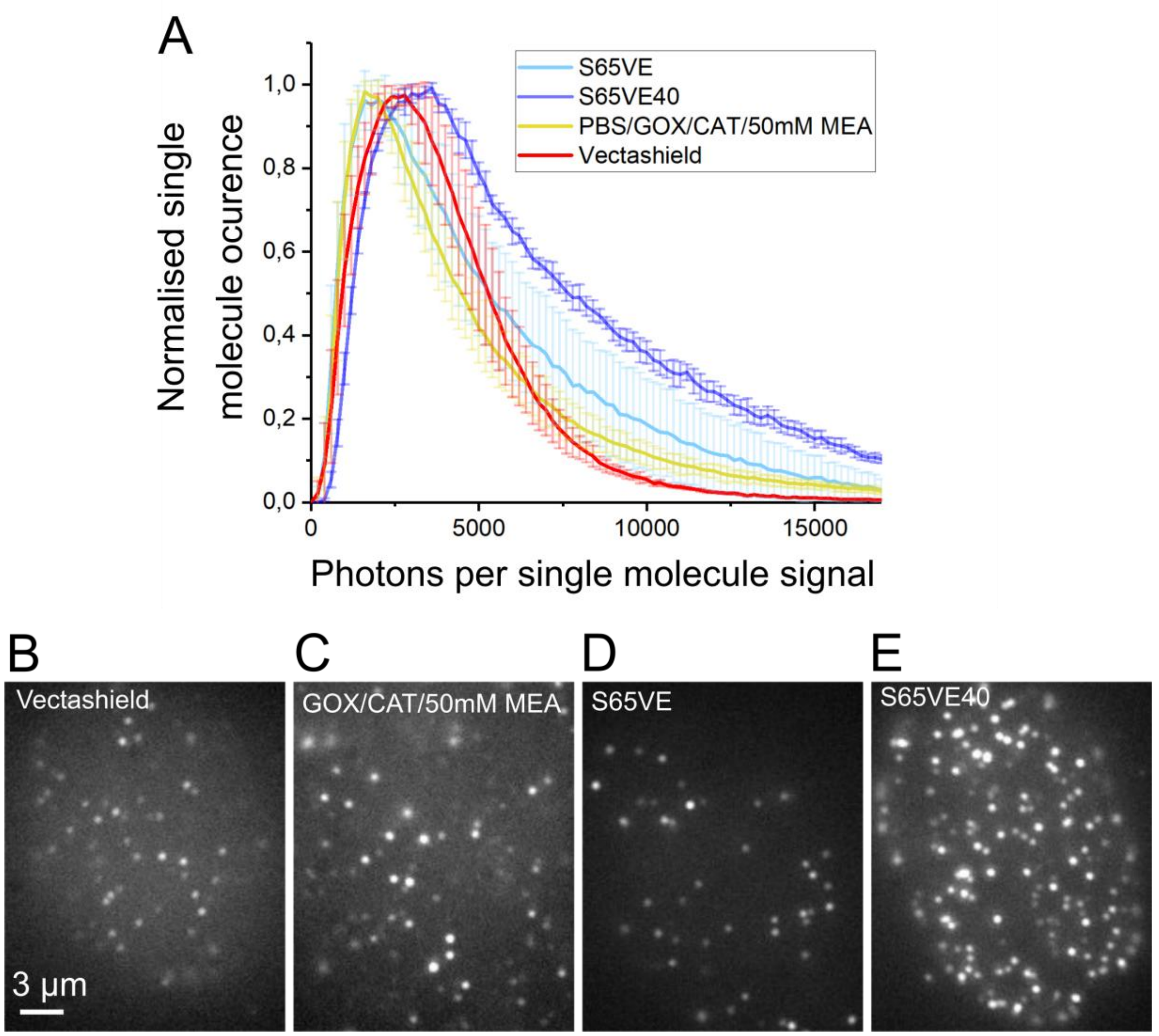

Supplementary Figure S14. Single Alexa 647 molecule photon counts can be increased by means of SIM-dedicated mounting media with varied composition. A) Distributions of photons emitted by individual Alexa 647 molecules conjugated to anti-Lamin B1 antibody and introduced to A549 adherent cells. Curves for Vectashield, standard PBS-based medium containing 10% (w/v) glucose oxidase (0.5 mg/ml), catalase (40 µg/ml), 50 mM cysteamine (Heilemann et al. 2008), S65VE (containing 20% vol/vol Vectashield), and S65VE40 (containing 40% vol/vol Vectashield) are presented. The curves and standard deviation represent mean from at least 5 independent measurements for each mounting medium. Examples of raw 25 ms frames from SMLM-movies obtained in Vectashield (**B**), GOX/CAT/MEA (**C**), S65VE (**D**), and S65VE40 (**E**). For more information on this experiment see **Figure 4**.

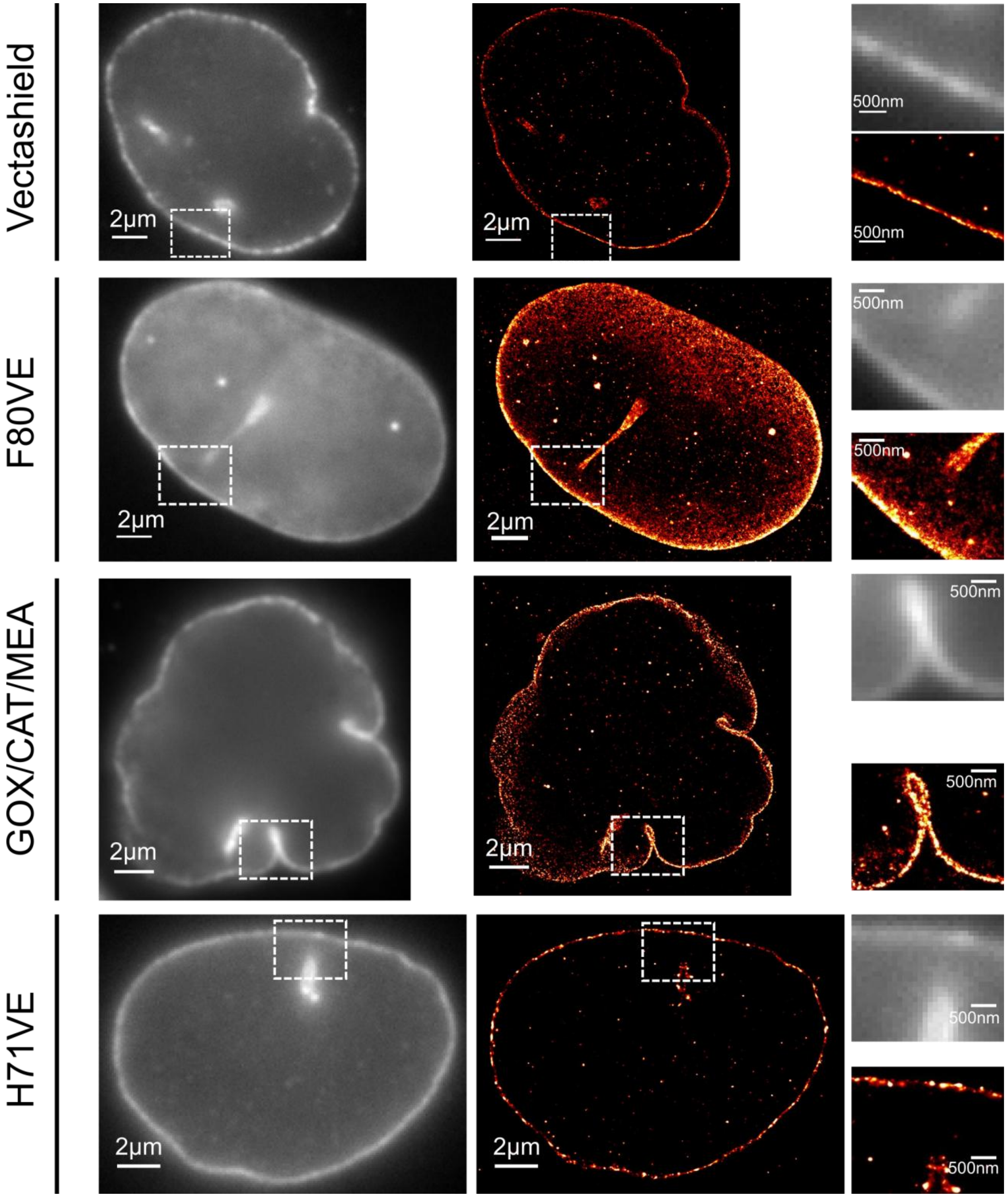

Supplementary Figure S15. SMLM reconstructions of Lamin B1-Alexa 647 embedded in various SIM-dedicated mounting media. Conventional wide-field images presented in grey. The images obtained in F80VE are of comparable quality to the ones obtainable in Vectashield. Images obtained in H71VE suffer from poor single molecule localisation density and poor photon yield (see **Fig. 4B** for more details). See Fig. 4E-F for respective images in S65VE and Sorb70VE. Image in standard glucose oxidase / catalase –based medium with addition of a thiol demonstrated for comparison (Heilemann et al., 2008)

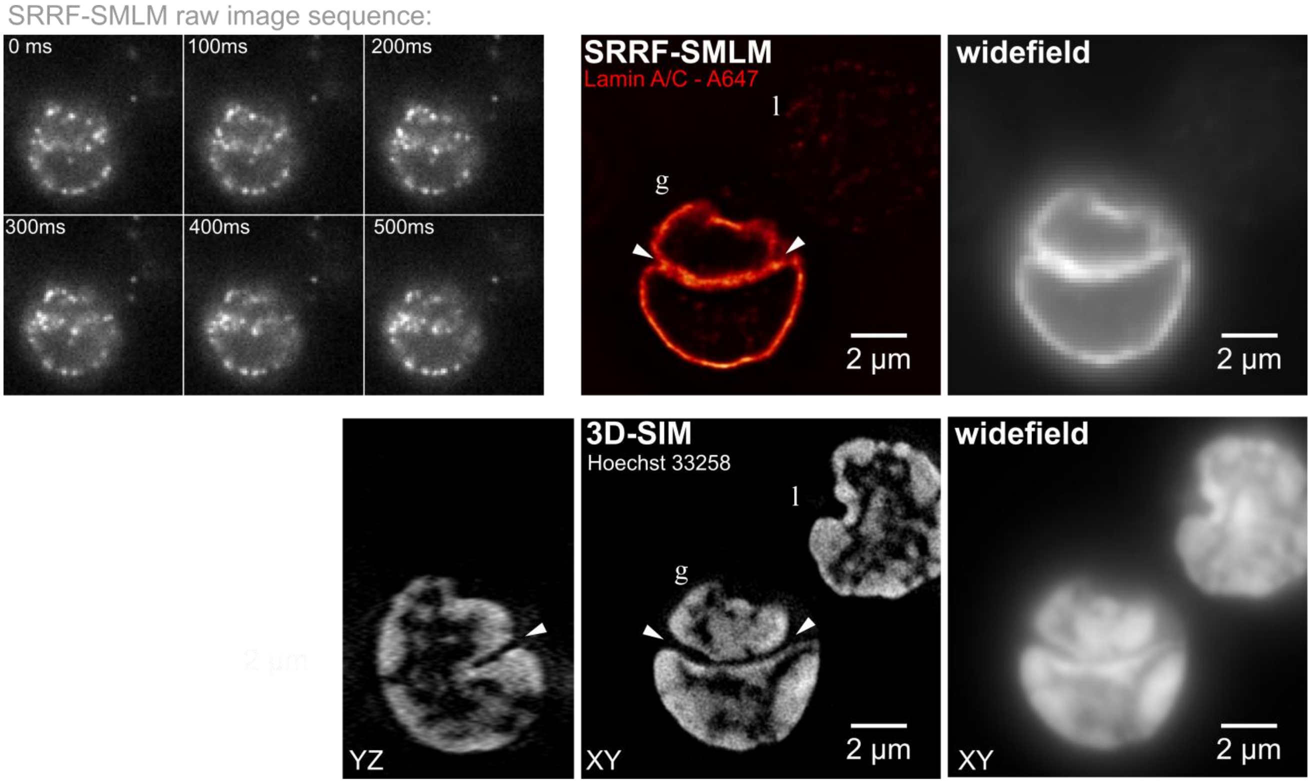

Supplementary Figure S16. Correlative 2D-SMLM & 3D-SIM of samples embedded in the SIM-dedicated mounting media. The figure demonstrates correlative super-resolution imaging of a lymphocyte (l) and a granulocyte (g) cells stained for Lamin A/C with Alexa647 (SRRF-SMLM, upper row) and for nuclear DNA using Hoechst 33258 (3D-SIM, lower row). The sample was freshly embedded in S65VE mounting medium containing 20% vol/vol Vectashield.S65VE medium necessitates moderately high exciting light intensity to induce blinking (fluctuation) of Alexa 647 signal (~0.2 kW/cm^2). This is required for super-resolution radial fluctuation SRRF-SMLM reconstruction of high-density data (Gustafsson et al. 2016). Conventional images corresponding to SRRF-SMLM and 3D-SIM images are presented in the right column, upper and lower row respectively. An example of a raw image sequence for SRRF-SMLM measurements is presented in the upper-left corner. This embedding procedure enables correlative SRRF & SIM using a commercial super-resolution microscopy set-up (see Materials and Methods for details). Arrowheads point at folding in granulocyte's nuclear periphery enriched in Lamin A/C. The wrinkle becomes clear when inspecting an orthogonal view (YZ) through a 3D-SIM image of the DNA (presented on the left).

**Mounting medium**	**Composition**<br>**Embedding procedure**	**Reference to first use of an agent:**	**Additional comments**
**Vectashield**	Vectashield H-1000 (Vectorlabs),<br>directly applied onto a sample	(Schermelleh et al. 2008)	standard non-hardening mountant for SIM
**S65VE**	0.8x 65% wt/wt sucrose/PBS + 0.2x Vectashield,<br>sample incubated in 30% wt/wt sucrose/PBS for 15min and directly transferred to S65VE	(Tsai et al. 2009)	Non-hazardous
**Sorb70VE**	0.8x 70% wt/wt sorbitol/PBS + 0.2x Vectashield,<br>sample incubated in 35% wt/wt sorbitol/PBS for 10 min and directly transferred to Sorb70VE	Sorbitol-based refractive index matching solution (sRIMS)<br>(Marx 2014)	Non-hazardous, dissolvable at 75% in H_2O
**F80VE**	0.8x 80% wt/wt fructose/PBS + 0.2x Vectashield,<br>sample incubated for 30min in 40% wt/wt fructose/PBS @ 37^oC and in F80VE for 30min @ 37^oC	See deep into brain (SeeDB)<br>(Ke, Fujimoto, and Imai 2013)	Non-hazardous, hard to dissolve at >80% wt/wt
**H71VE**	0.8x 71.4% wt/wt Histodenz/PBS + 0.2x Vectashield,<br>30min in 40% wt/wt Histodenz/PBS @ 37^oC,<br>30min in H71VE @ 37^oC	Refractive index matching solution (RIMS)<br>(Yang et al. 2014)	Non-hazardous, dissolvable at ~80% wt/wt
**TDE97**	0.97x 2,2'-thiodiethanol + 0.03x PBS,<br>5 min in 10, 25, 50, 97% TDE, each	(Staudt et al. 2007)	hazardous, decreases sample hydration

Supplementary Table 1. Composition of SIM-dedicated mounting media tested in this study. Wt/wt dilutions of substances were prepared in 1x PBS. Vol/vol dilutions were used elsewhere, e.g. when 0.8x 65% wt/wt sucrose + 0.2x Vectashield is stated it means that 800 µl of 65% wt/wt sucrose solution in PBS together with 200 µl of Vectashield were mixed. Solutions of sugars, sugar alcohols and Histodenz were prepared in advance through repeated rapid stirring and warming up. Vectashield was added to the media immediately prior to the sample embedding. In experiments presented in **Supplementary Figure S10** 20% vol/vol of Vectashield was replaced with 0 and 60% vol/vol concentrations.

Mounting medium:	Refractive index @ 25°C, 589 nm	Time to fill 130 µl pipette tip (viscosity measure) [sec]
30% wt/wt sucrose / PBS	1.3890	<1
65% wt/wt sucrose / PBS	1.4594	12
35% wt/wt sorbitol / PBS	1.3991	<1
70% wt/wt sorbitol / PBS	1.4609	8
40% wt/wt fructose / PBS	1.4100	<1*
80% wt/wt fructose / PBS	1.4818	48*
100% glycerol	1.4718	52
40% wt/wt Histodenz / PBS	1.4174	<1*
71.4% wt/wt Histodenz / PBS	1.5163	12*
97% vol/vol 2,2'-thiodiethanol / PBS	1.5149	3
Vectashield	1.4477	6

Supplementary Table 2. Measurements of refractive indices and assessment of viscosity for various combinations of media for tissue clearing. Note very low viscosity of the intermediate concentrations. Lower concentrations of clearing agents can be utilised if a decreased viscosity and an increased sample penetration is required. For instance, good SIM results were also obtained with Sorb35VE medium based on only 35% wt/wt sorbitol solution. Viscosity was estimated using a "pipette tip assay"; 130 µl pipette tip was used to measure time required to completely fill it up. A similar approach was previously used (Hou et al. 2015). For all of the media the viscosity decreased when temperature was elevated to 37°C. Note that the embedding in some mounting media dedicated for SIM experiments was performed at 37°C (asterisk, for the detailed procedures see **Supplementary Table 1**).

Medium	Fluorescence MIN	Fluorescence MAX	Dynamic Range
Hodgkin's lymphoma cells (Figure 1, 2, Supplementary Figures S3, S4)			
**S65VE**	4408(521.9)	52440(9046.2)	12.4(2.6)
**Sorb70VE**	2736(304.6)	59960(12312)	21.4(4.2)
**F80VE**	5016(443.0)	52592(9282.0)	10.2(1.6)
**H71VE**	2296(119.5)	28296(16284.6)	12.9(6.3)
**Vectashield**	5244(290.9)	18552(1847.6)	3.6(0.3)
10 µm–thick mouse spleen tissue sections (Figure 3, Supplementary Figure S7)			
**S65VE**	7740(2859.4)	34848(16710.9)	4.4(0.8)
**Sorb70VE**	13544(4402.0)	65532(0)	5.3(0.8)
**F80VE**	9256(1740.2)	59501(6363)	6.6(0.9)
**H71VE**	12538(219.1)	65490(71.6)	5.2(0.1)
**Vectashield**	7485(2333.6)	33170(4760.0)	4.6(1.2)

Supplementary Table 3. Comparison of the fluorescence signal intensity in raw 3D-SIM images of Hoechst 33258-labelled HL cells and 10 µm-thick mouse spleen sections. Median minimal and maximal pixel intensities together with corresponding median dynamic range (max/min ratio) are presented in the table. Median values were calculated from many individual measurements. Standard deviation is given in brackets. Numbers for HL data set for 1.512 immersion oil is presented. Note a large difference in minimal and maximal signal intensity for all the media between HL cells and mouse spleen tissue sections. Note that the dynamic range values obtained in mouse spleen sections do not correlate with MCNR values presented in **Figure 3F**.

References

Ball, Graeme et al. 2015. “SIMcheck: A Toolbox for Successful Super-Resolution Structured Illumination Microscopy.” *Scientific Reports* 5(1): 15915. http://www.nature.com/articles/srep15915.

Gustafsson, Nils et al. 2016. “Fast Live-Cell Conventional Fluorophore Nanoscopy with ImageJ through Super-Resolution Radial Fluctuations.” *Nature communications* 7: 12471. http://www.ncbi.nlm.nih.gov/pubmed/27514992.

Heilemann, Mike et al. 2008. “Subdiffraction-Resolution Fluorescence Imaging with Conventional Fluorescent Probes.” *Angewandte Chemie (International ed. in English)* 47(33): 6172–76. http://www.ncbi.nlm.nih.gov/pubmed/18646237 (January 23, 2014).

Hou, Bing et al. 2015. “Scalable and DiI-Compatible Optical Clearance of the Mammalian Brain.” *Frontiers in neuroanatomy* 9(February): 19. http://journal.frontiersin.org/Article/10.3389/fnana.2015.00019/abstract.

Ke, Meng-Tsen, Satoshi Fujimoto, and Takeshi Imai. 2013. “SeeDB: A Simple and Morphology-Preserving Optical Clearing Agent for Neuronal Circuit Reconstruction.” *Nature Neuroscience* 16(8): 1154–61. http://www.nature.com/doifinder/10.1038/nn.3447.

Marx, Vivien. 2014. “Microscopy: Seeing through Tissue.” *Nature Methods* 11(12): 1209–14. http://dx.doi.org/10.1038/nmeth.3181.

Ovesný, Martin et al. 2014. “ThunderSTORM: A Comprehensive ImageJ Plug-in for PALM and STORM Data Analysis and Super-Resolution Imaging.” *Bioinformatics (Oxford, England)* 30(16): 2389–90. http://www.ncbi.nlm.nih.gov/pubmed/24771516 (September 29, 2014).

Schermelleh, Lothar et al. 2008. “Subdiffraction Multicolor Imaging of the Nuclear Periphery with 3D Structured Illumination Microscopy.” *Science (New York, N.Y.)* 320(5881): 1332–36. http://www.pubmedcentral.nih.gov/articlerender.fcgi?artid=2916659&tool=pmcentrez&rendertype=abstract.

Staudt, Thorsten et al. 2007. “2,2′-Thiodiethanol: A New Water Soluble Mounting Medium for High Resolution Optical Microscopy.” *Microscopy Research and Technique* 70(1): 1–9. http://www.ncbi.nlm.nih.gov/pubmed/7068228.

Tsai, P. S. et al. 2009. “Correlations of Neuronal and Microvascular Densities in Murine Cortex Revealed by Direct Counting and Colocalization of Nuclei and Vessels.” *Journal of Neuroscience* 29(46): 14553–70. http://www.jneurosci.org/cgi/doi/10.1523/JNEUROSCI.3287-09.2009.

Yang, Bin et al. 2014. “Single-Cell Phenotyping within Transparent Intact Tissue through Whole-Body Clearing.” *Cell* 158(4): 945–58. http://dx.doi.org/10.1016/j.cell.2014.07.017.
